# Runaway electrons and their interaction with tungsten wall: a comprehensive study of effects

**DOI:** 10.1038/s41598-023-48672-7

**Published:** 2023-12-08

**Authors:** Laleh Ataeiseresht, Mohammad Reza Abdi, Banafsheh Pourshahab, Chapar Rasouli

**Affiliations:** 1 Faculty of Physics, University of Isfahan, Isfahan, Iran; 2 Plasma and Nuclear Fusion Research School, Nuclear Science and Technology Research Institute (NSTRI), AEOI, Tehran, Iran

**Keywords:** Nuclear fusion and fission, Magnetically confined plasmas

## Abstract

Runaway electrons are a notable phenomenon occurring during the operation of a tokamak. Proper material selection for the tokamak's first wall structure and plasma facing components, particularly in large sizes tokamaks like ITER and DEMO, is crucial due to the energy deposition of runaway electrons on plasma facing components during collision events, resulting in severe heat transfer and material damage in the form of melting, corrosion, and fracture. These runaway electrons also contribute to the production of photoneutrons through (γ, n) nuclear reactions, lead to material activation and require remote handling. In this study, using a Monte Carlo code and simulating the collision of runaway electrons with a tungsten target exposed to their radiation, the electron transport is investigated, and the energy deposition spectrum resulting from these collisions on the target is analyzed. The influence of incident angle and magnetic field on the energy deposition spectrum and the energy deposition per particle in the target is examined. With an increase in the incident angle of incoming electrons, the amount of energy deposited in the target rises and the energy deposition spectrum broadens. Moreover, applying a magnetic field, results the most significant increase in energy deposition for electrons with energies below 1 MeV in the tangential radiation case. The energy deposition spectrum resulting from each collision event in these interactions is determined. For electrons with energies below 5 MeV, multiple scattering and ionization processes are the primary contributors to energy deposition in the target. However, as the incident electron energy increases, the significance of multiple scattering and ionization diminishes, and the bremsstrahlung process becomes the most effective reaction in energy deposition. The energy deposition profile of electrons in the tungsten target indicates that higher incident electron energies lead to a shift of the maximum energy deposition location towards the inner layers of the target, and the energy deposition peak broadens. Analyzing the electrons transport inside the tungsten target reveals that a substantial portion of electrons with energies of 50–100 MeV passes through the wall and may exit from the back surface, potentially causing damage to equipment behind the tungsten wall. Additionally, secondary products of the reaction, such as photons, secondary electrons, and neutrons and their energy profiles are thoroughly studied. These secondary products can penetrate the target and activate materials in the equipment behind the plasma-facing components. For primary electrons below 1 MeV hitting tungsten, reflection process is significant. Analysis of primary and secondary runaway electrons in the tokamak's tungsten wall shows that electrons with energies of 0.1, 0.2, and 0.5 MeV predominantly interact within a first 0.1 mm layer, without passing through it. The secondary electrons can escape the tungsten target and impact other components, which making them an important consideration in runaway electron collisions with the tokamak wall. Produced photons, as one of the secondary products, also linearly increase with the rising energy of primary electrons. Also, the photoneutrons are produced only when runaway electrons with energies of 10 MeV and above collide with the target. These secondary products can penetrate the target and activate materials in the equipment behind the plasma-facing components.

## Introduction

The phenomenon of runaway electrons is one of the phenomena occurring during tokamak operations. Runaway electrons have been found since the beginning of tokamak physics^1–3^. Energy deposition of plasma particles on plasma facing components is one of the fundamental physical processes in confinement loss events such as disruptions^4^.

One of the issues that researchers have long time involved in is the harmful consequences of runaway electrons on the operation of nuclear fusion reactors. For example, the 1976 report on the effect of large fluxes of runaway electrons in the TFU 400 and Alcator tokamaks which led to the creation of holes in the wall of the vacuum vessel^5^ or the 1981 report in which the graphite damage of Doublet-III tokamak limiter caused by runaway electrons have been investigated^6^, or in 1994 the Tore Supra limiter pump was damaged by runaway electrons during a disruption and led to water leakage and material activation^7^. All these events emphasize that runaway electrons can be produced in different phases during a tokamak operation^8^. In other words, runaway electrons can be produced during the start phase (in tokamak plasmas), in Ohmic discharges at low densities, and in the phase of current quench in the disruptions. The most dangerous situation occurs in disruption when runaway electrons with energy of several tens of mega electron volts are produced, or the runaway electrons' contribution to the formation of plasma current is more than sixty percent^9^. Since high-energy electrons have the potential to collide with vacuum vessel wall and deposit their energy locally, which result in damage to the wall material, in large fusion machines it is tried to prevent the production of runaway electrons as much as possible^10,11^. For this purpose and with the aim of preventing disruption and its consequences, including reducing the risks associated with the effects of runaway electron beams, many efforts have been made and new solutions are being developed and tested to enable the successful operation of ITER^12,13^.

A key impediment to an effective magnetic fusion energy concept is performance during abnormal events including plasma disruption and edge-localized modes (ELMs) due to loss of plasma confinement^14^. Tokamak plasmas are inherently very sensitive to certain operating conditions. For example, if the electron density or plasma current is too high, or if heavy impurity atoms accumulate too much in the plasma, the energy confinement may be severely reduced and lead to disruption^15,16^. During the disruption, at first, the plasma temperature drops significantly in a short time (usually in the order of milliseconds), which is known as the thermal quench phase^15^. The decrease in temperature causes an increase in collisions between particles, which leads to a significant increase in plasma resistance and severely limits the ability of plasma to carry the toroidal current. In the second stage, the plasma current is terminated and this state is performed in a time that is usually longer than the thermal quench time (around 10 ms). This phase is called the current quench phase. According to Faraday's law, the plasma tends to maintain the poloidal flux and this leads to the generation of a strong toroidal electric field, thus the decay of the plasma current must create an induced electric field that is proportional to the rate of current decay^17^. Due to the high decay rate of plasma current, the induced electric field reaches high values, in which the normal electrons of the plasma can be strongly accelerated and reach high energies^18^ The effect of runaway electrons that hit the vessel Walls, strongly depends on the energy obtained in the toroidal electric field of the tokamak^19^. Plasma instabilities such as hard disruptions and ELM, because of their short durations, will mainly cause surface damage to plasma-facing materials. Surface damage includes high erosion losses from vaporization, spallation, and melt-layer erosion^20^.

As mentioned above, when the disruption occurs, increasing the force on electrons from the toroidal electric field against the friction force of the background ions causes the electrons accelerate to relativistic velocities and leave the distribution function. This mechanism is known as Dreicer mechanism. In addition, other mechanisms of runaway electron production are the hot tail mechanism and the avalanche mechanism^21,22^. Based on calculations and simulations, it is expected that in large-sized tokamaks such as ITER, during the current quench phase related to disruption, a large current of runaway electrons will be generated and according to the available references, the maximum energy of runaway electrons in ITER is expected to be in the range of 1–100 MeV^23–25^.

Therefore selection of suitable materials for the structure of the tokamak wall and plasma facing components is especially important in large size tokamaks such as ITER and DEMO because at the moment of collision, the energy of runaway electrons in the time range of milliseconds is deposited on the surfaces of plasma facing components and the intense heat transfer causes damage to the materials in the form of melting, corrosion and even breakage of the plasma facing components. These electrons also lead to the production of photoneutrons through nuclear reactions (γ, n)^26^. Therefore, material activation occurs and creates the need for remote maintenance. Highly localized and deep thermal damage of plasma facing components due to collision with runaway electrons also creates costly repairs and, more importantly, the need for long shutdown of the device^18,27^.

ITER has implemented a fully tungsten (W) armored divertor, with tungsten selected as the material for plasma-facing components, including the dome, outer vertical target, and inner vertical target in the form of monoblocks or flat tiles^28^. It can withstand high power density and a certain number of unplanned plasma-wall contacts at high current due to a robust first wall capable of enduring stationary phases. In DEMO, higher neutron fluence on plasma-facing components, driven by increased fusion power and longer required lifetime, requires material adaptation. The first wall in DEMO is thinner than ITER's to facilitate Tritium breeding, allowing neutron streaming across Plasma Facing Components (PFC) into the breeding region. Due to these considerations, the DEMO design incorporates high heat flux 'sacrificial' limiters^29,30^. The baseline model for divertor target plate plasma-facing units is the ITER-type monoblock design^31^.

Therefore, the need to study the destructive effects of runaway electrons and damage caused by runaway electrons on the plasma facing components, including the first wall and the divertor and, if available, the limiter, is one of the most important issues related to the design of fusion reactors. In this study, by simulating the impact of runaway electrons on the targets that are exposed to the radiation of these electrons, using a Monte Carlo code, the transport of electrons is investigated and the energy deposition spectrum caused by this electron beam on the target is extracted. Then, by obtaining the spectrum of energy deposition caused by each phenomenon occurring in this collision, the contribution of each of these phenomena to the spectrum of total energy deposition has been investigated. From this study, it is clear how the contribution of each of these phenomena in energy deposition will change with the change of electron energy. In addition, the effect of radiation angle and magnetic field on the spectrum of energy deposition and the amount of energy deposition per particle in the target has been examined. After acquiring the energy spectrum and determining the energy deposition per particle on the target, a study conducted to analyze the amount of energy deposition and its variations at different depths within the target material. In the second step, the penetration depth of the incident electrons in the target was analyzed with the energy variation. In this regard, the quantity of incident electrons that penetrated through the target, their reflection from the initial impact surface, and their passage through the target's end surface was investigated. Furthermore, this analysis was extended to include the reaction products resulting from the collision of electrons with the target, such as secondary electrons, neutrons, and photons. The investigation aimed to identify additional effects of high-energy electron collisions on the target, which serves as a model for the tokamak wall.

## Configuration and analysis procedures

In this section, the model considered for simulation, will be described in order to investigate the effect and penetration depth of the runaway electrons on the plasma facing components and the tokamak wall, which include the first wall and the divertor.

### Plasma facing components configuration and loading conditions

**Figure 1:**
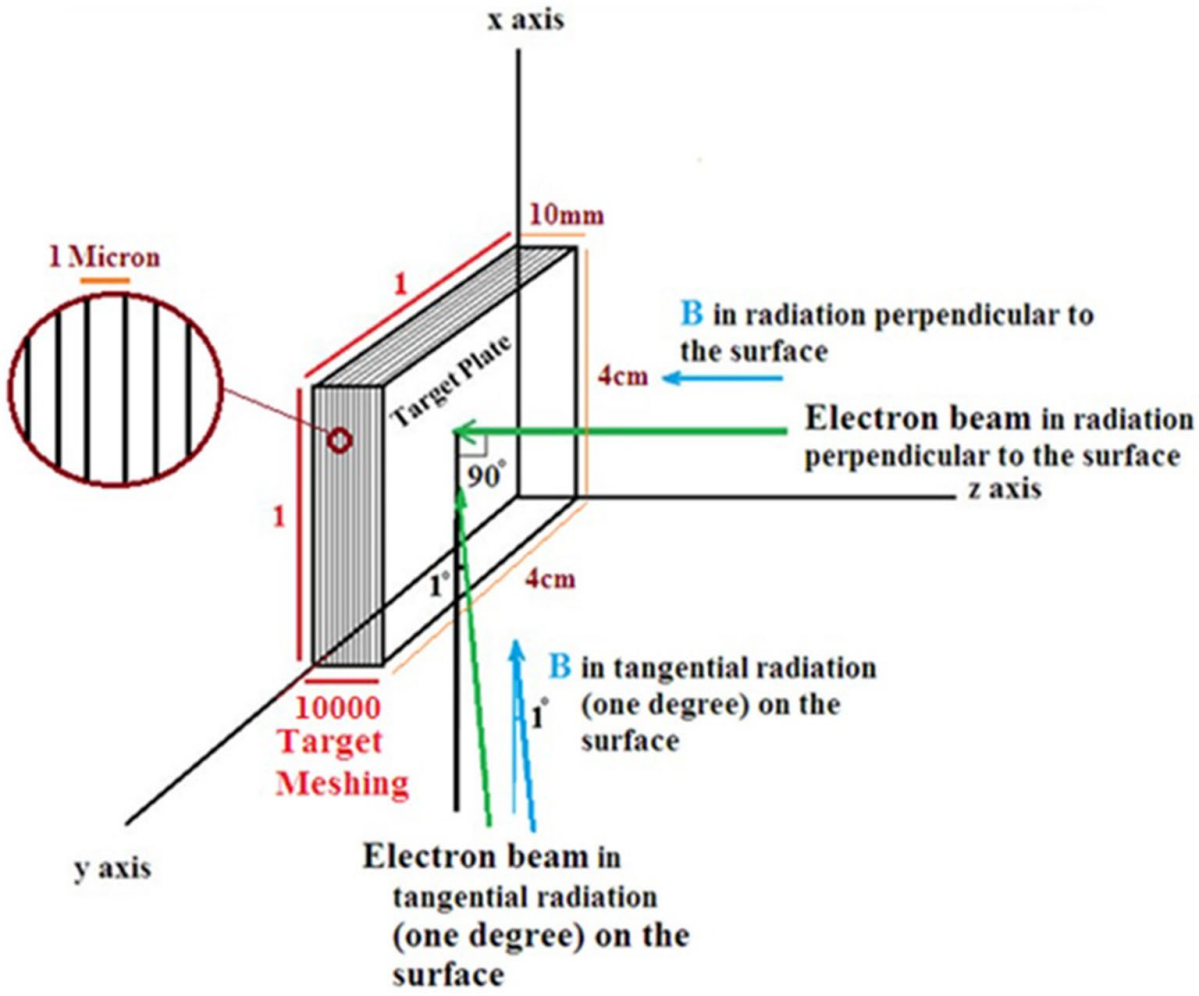
Tungsten target meshing in order to obtain energy deposition at the depth of the target and how the electron beam is irradiated perpendicularly and tangentially to the tungsten target.

A square target with a cross-sectional area of 40 mm × 40 mm and a thickness of 10 mm is considered for the calculations. The reason for choosing this thickness is that for a tokamak like ITER, the thickness of the tungsten wall in the divertor area is 13 mm and in the armor section of the plasma facing components is 6 mm^32–34^. This value is in the range of selected thicknesses for tungsten in large size tokamaks. The diameter of the runaway electron beam is considered to be 3 mm, and the angle of impact of the electrons with the target has been investigated in both perpendicular (angle of 90-degree to the target) and tangential (angle of 1 degree to the target) modes. The manner of radiation of the electron beam to the target in the tangent and perpendicular state, as well as the direction of the magnetic field, if applied, is shown in Fig. 1. Considering that the maximum energy of runaway electrons in the ITER is predicted to be 1–100 MeV^24,25^, In this research we tried to use a wide range of energy in the simulation and used low energy electrons (0.1 MeV) and also high energy electrons of 50 MeV and 100 MeV in the calculations to consider this purpose. Therefore, single-energy incident electrons for 10 different energies of 0.1, 0.2, 0.5, 1, 2, 5, 10, 20, 50 and 100 MeV are considered in the calculations. It is assumed that the target is located in the x–y coordinate plane and the electron beam is irradiated along the z axis. The target material chosen for this study is tungsten. Tungsten metal has unique properties such as high melting point, high resistance to sputtering and neutron damage, low activation rate against neutron radiation, low vapor pressure at melting temperature, low corrosion and good thermodynamic properties; Therefore, among the existing candidates, it is a main selection for use as plasma facing components and armour in the divertor of fusion reactors such as ITER and DEMO and also as the material used in limiters of DEMO^35^. In order to perform calculations in each step, the number of particles hitting the target is considered five million.

### Computational tools

In this research, the Monte Carlo Geant4 code was used to perform the calculations, and the transport of runaway electrons in the tungsten target was investigated using it. Geant4 is a Monte Carlo code used to simulate high-energy physics detectors at CERN. This code also calculates electromagnetic processes for electrons and photons with energies from hundreds of Kilo electron volts (keV) to several hundred Tera electron volts (TeV). Geant4 programming tool provides a detailed library of physical processes includes particle-matter interactions and is widely used in particle physics, medical studies, and space science^36^. Depending on the energy regime in which the simulations are performed, Geant4 provides several models in the form of a physics list that includes the required physical processes. In this study, in the first part of the simulation, which includes the calculation of the spectrum of energy deposition, the contribution of energy deposition caused by each process in the target per particle, and the calculation of the total energy deposition in the depth of the target, the processes of gamma conversion, multiple scattering, bremsstrahlung, ionization, Compton scattering, photoelectric, pair production and pair annihilation have been considered and the amount of energy deposition due to each of these phenomena has been calculated for a particle hitting the target. In the second part, hadronic physics was added to enhance knowledge about the penetration behavior of primary electrons and the behavior of secondary products resulting from electron collisions with tungsten. This included investigating the direct interactions of photons with the tungsten nucleus, characterizing neutron behavior, and exploring gamma-to-neutron conversion reactions and photoneutron production. These additions enriched the understanding of electron-tungsten interactions presented in the first part. It is necessary to clarify that the results showed that adding hadron physics does not have a significant effect on the amount of energy/particle deposition and only investigates the reactions in more detail. In order to obtain the amount of energy deposition in the depth of the target (in the direction of the z axis), considering the one-dimensionality of the problem, by doing the meshing in target, the energy deposited due to all the interactions of the electron with the target in each mesh is obtained. The amount of particle energy deposition in the target depth is calculated for a single energy electron beam with different energies. Target meshing is done as shown in Fig. 1. The length and width of the target in the x–y plane is divided into one part and its thickness along the z axis is divided into 10,000 parts. Considering the thickness of the target, which is 10 mm, actually the meshes are plates parallel to the main plate and have a thickness of one micron.

## Result and discussion

In the simulations conducted in this research, two main sections were examined. The first section involved calculating the energy deposition of electrons in the target. Initially, the energy deposition spectrum of all electrons in the target was computed, and the effects of magnetic fields and the angle of electron incidence on the energy deposition spectrum were investigated. Subsequently, the contribution of each process to the overall energy deposition spectrum was examined, and variations in this contribution were studied concerning electron energy variation and the remaining energy of each electron for each process in the target. In second section, the amount of electron energy deposition for all processes in the depth of the target has been obtained. In this regard, the amount of production of secondary particles in the target, their energy and the manner of their propagation in the target were obtained and the secondary reactions that may cause neutron activation were investigated.

### Investigating the energy deposition spectrum of electrons in the target

By simulating transport of electrons within the target, the total energy deposition spectra for energies 0.5, 1, 2, 5, 10, 20, 50 0.1, 0.2, and 100 MeV were calculated in different states and each of these scenarios will be explained.

#### Energy deposition spectrum for electron beam at 90-degree incidence to the target

**Figure 2:**
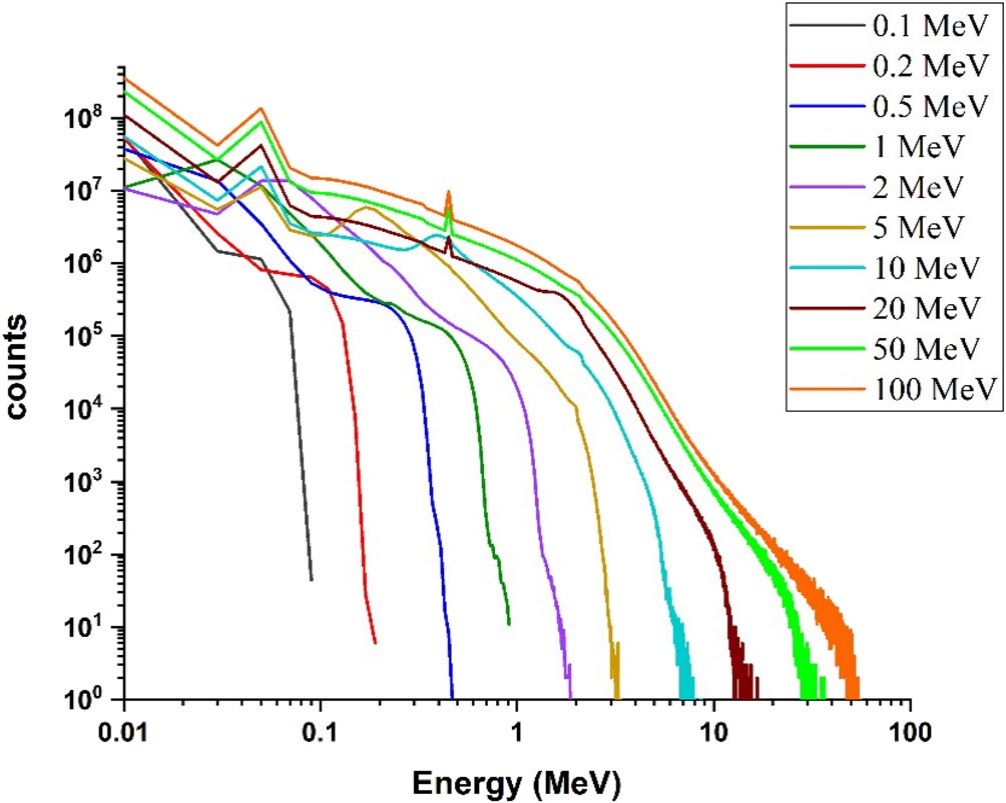
Spectra of deposited energy in the target corresponding to the perpendicular radiation mode of single energy electron beam, to tungsten target for energies 0.1–100 MeV.

Using the Geant4 code, the energy deposition spectrum of electrons in the target, which is actually the abundance of energies deposited during the interaction of single-energy electron beams in radiation perpendicular to the tungsten target surface (90° angle with surface of target as Fig. 1) with a thickness of 10 mm, for electrons with energy 0.1, 0.2, 0.5, 1, 2, 5, 10, 20, 50 and 100 MeV were calculated; the results are shown in Fig. 2.

In this study, the particles hitting the target are single-energy, but as shown in Fig. 2, in the energy deposition diagram, a range of energies absorbed in target is observed. The reason is that these electrons interact with the target particles after the collision, and as a result of these interactions, their energy level decreases, and energy absorption in amounts lower than the primary electron energy is also possible. According to the number of electrons hitting the target, eventually there will be a possibility of deposition in the entire energy range. In this simulation, the amount of energy absorbed in the target is recorded from the moment of electron collision until it comes to rest, with five million collisions counted. The energy deposition spectrum is presented in a graph to demonstrate the distribution of absorbed energies. As seen in the figure, for electron beams with low energies, the energy deposition process continues from 0 to the incident electron energy; for example, for a 0.1 MeV incident electron, the energy deposition range continues up to 0.1 MeV. However, with the increase in the energy of the electron beam, the range of energy deposition in the target is narrowed; for example, energy deposition range is up to 0.75 MeV for 1 MeV electrons, 7 MeV for 10 MeV electrons, and 50 MeV for 100 MeV electrons. This decrease can be attributed to the increase in the penetration depth of the incident electron in the target due to its high energy and the reduction in the probability of the interaction of electrons in the target, and as a result, the deposited energy decreases. Since the penetration depth of electrons with 100 MeV energy is approximately 10 mm, by choosing a thickness of 10 mm, some of the incident electrons will have a chance to pass through the target without interacting with it. Furthermore, the limited thickness of the target causes a significant number of secondary particles to escape from it. Consequently, for this 10 mm thickness, the range of energy deposition for high-energy electrons will be narrower than that for low-energy electrons. As it can be seen, up to 10 MeV energy, the amount of energy deposited from each incident electron in the target increases to about 80% of the total energy of the electron. Nevertheless, at higher energies, this amount decreases compared to the energy of the incoming particle, so that for an electron with energy of 100 MeV, this value reaches approximately 50% of the total input energy. The reason for this can be attributed to the finite thickness of the target compared to the penetration depth of higher-energy electrons. This finite thickness reduces the probability of interactions with the target, and increase escape of some of the secondary particles produced during these interactions. In fact, the deposited energy per incident electron is obtained by averaging the total deposited energy to the number of particles, and for high-energy electrons, due to the large penetration depth of the particles compared to the thickness of the target, the particles pass through the target and leave less energy. Therefore, the total deposition energy and as a result the deposition energy caused by per particle decreases according to the incident electron energy, which can be seen in Fig. 2.

#### Investigation of the energy deposition spectrum of runaway electrons and electron penetration depth in target considering the impact angle

**Figure 3:**
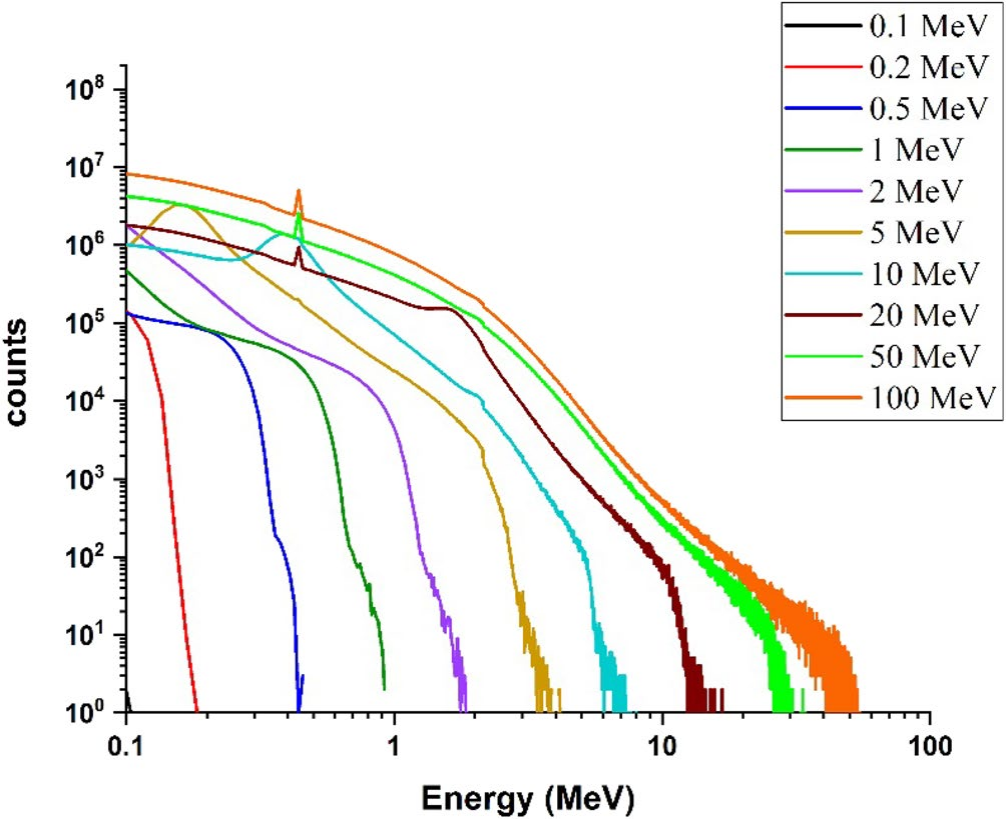
Spectra of deposited energy in the target corresponding to the tangential radiation mode (one-degree angle) of single energy electron beam, to the tungsten target for energies 0.1–100 MeV.

In this section, assuming that the runaway electron beam hits the target tangentially (angle of 1 degree), the total energy deposition spectrum and the amount of energy deposition caused by each particle in the target have been calculated. The spectrum of energy deposition for tangential irradiation of the beam with the considered energies is obtained according to Fig. 3.

**Figure 4:**
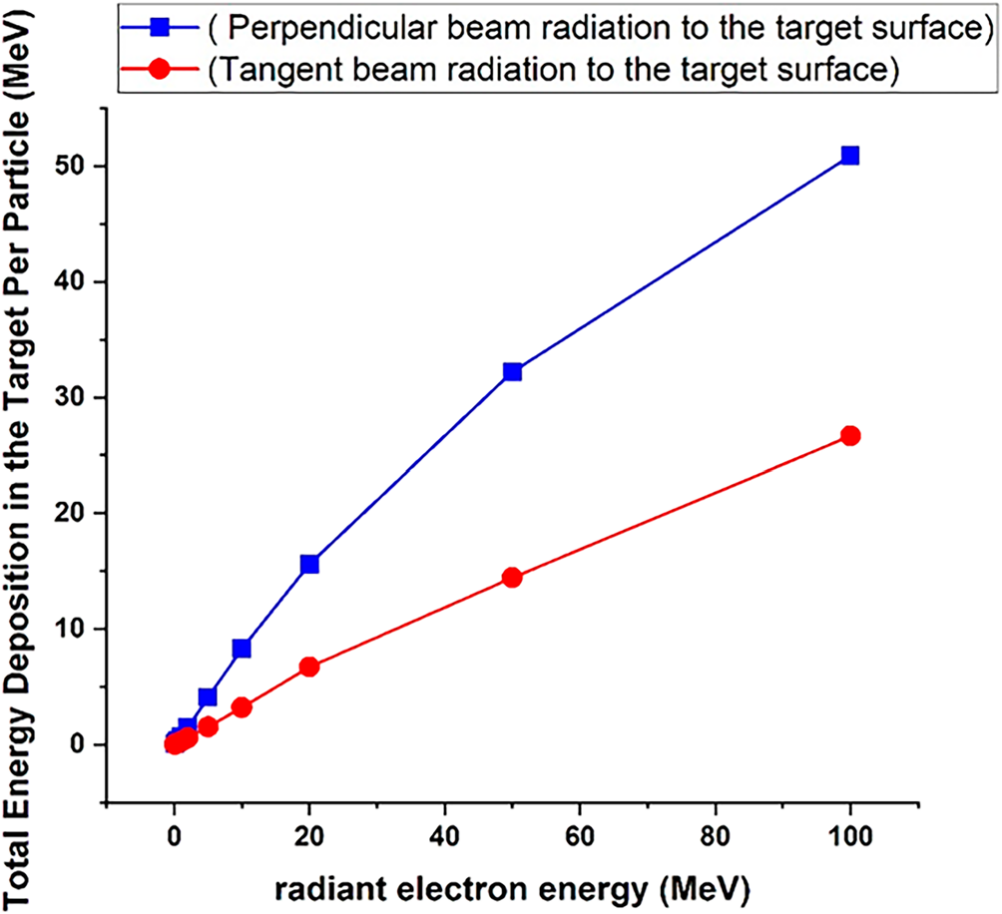
Energy deposited in the target due to the impact of the electron beam perpendicularly and tangentially to the target, in the absence of the magnetic field for per electron particle.

Comparison of Figs. 2 and 3 shows that by changing the beam angle of runaway electrons from perpendicular to tangent with tungsten target, the energy deposition range decreases by a very small amount. The abundance of deposited energies for low energy electrons has decreased slightly compared with the perpendicular radiation. The reason for this can be due to the increase in the scattering of electrons and their reflection in the tangential collision with the target. The amount of energy deposited in tungsten target with a thickness of 10 mm per incident electron in perpendicular and tangent radiation state is given in Fig. 4.

**Figure 5:**
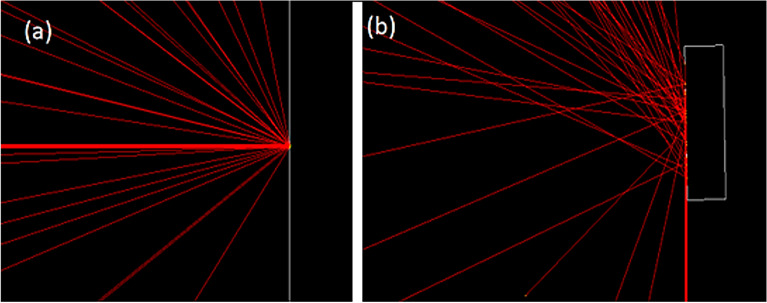
Beam radiation of 0.1 MeV electron to tungsten target (**a**) perpendicular and (**b**) tangentially.

By comparing the energy deposited per particles in Fig. 4, it is evident that the energy deposition per particle in the target for tangential radiation is approximately 40% of the value for perpendicular radiation for energies less than 50 MeV. However, for energies higher than 50 MeV, the energy deposition for tangential radiation is approximately 50% of the value for perpendicular radiation. Considering that in the case of tangential radiation, the amount of reflection of particles from the surface is higher; the amount of energy deposited in the target is also reduced. Fig. 5 shows the transport of 50 particles for an electron beam with energy of 0.1 MeV in two conditions of perpendicular and tangential radiation with tungsten target. As can be seen, in the case of tangential radiation the number of electrons reflected from the target surface is much higher than the case of electron beam perpendicular radiation on the surface of target.

The reason for this is related to the problem of particle scattering. When the electrons hit the target, some of them with a smaller impact parameter undergo large angle scattering ($\frac{\pi }{2}\le \theta \le \pi$) and electrons with a larger impact parameter undergo small angle scattering ($\theta \ll \frac{\pi }{2}$), which in the case of perpendicular radiation of electrons to the surface, Only in the case of large angle collisions, the particles are reflected and do not enter the target. But in the case of tangential impact, even particles with a scattering angle of less than 90 degrees have the possibility of reflecting outside the target, so more particles are reflected from the surface of the target in tangential mode^37^.

### Investigation of the energy deposition spectrum of runaway electrons and particle penetration depth in target in the presence of magnetic field

#### Perpendicular radiation and presence of a magnetic field in the direction of electron beam

**Figure 6:**
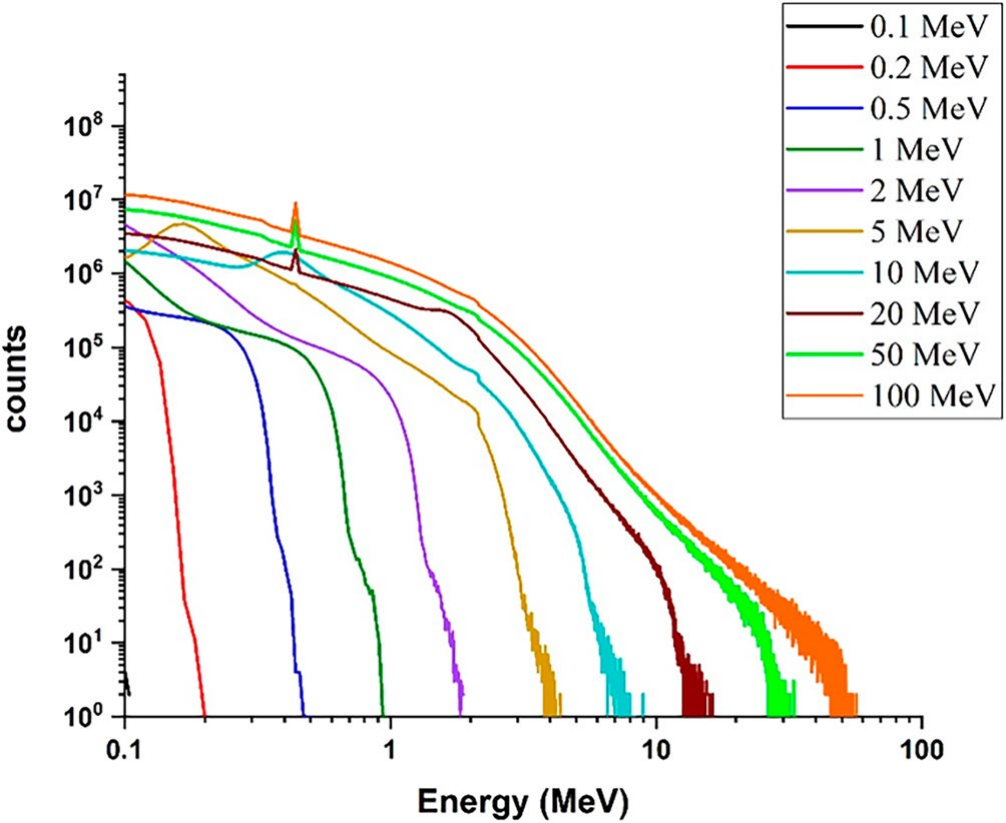
Spectra of deposited energy in the target corresponding to the perpendicular radiation mode of single energy electron beam, to tungsten target in the presence of a magnetic field 1 T for energies 0.1–100 MeV.

If we assume that 1 T magnetic field is applied in the perpendicular direction to the target (with an angle of 90 degree) and the single energy electron beam collides perpendicularly (in the direction of the z axis and parallel to the field) with the tungsten target in the vacuum environment, the energy deposition spectrum is obtained according to Fig. 6.

By comparing Fig. 6 (radiation perpendicular to the target surface in the presence of a magnetic field) and Fig. 2 (radiation perpendicular to the target surface without the presence of a magnetic field), it can be seen that the general trend of the diagram is the same and there are no reasonable differences between them.

#### Tangential radiation and presence of a magnetic field in the direction of electron beam

**Figure 7:**
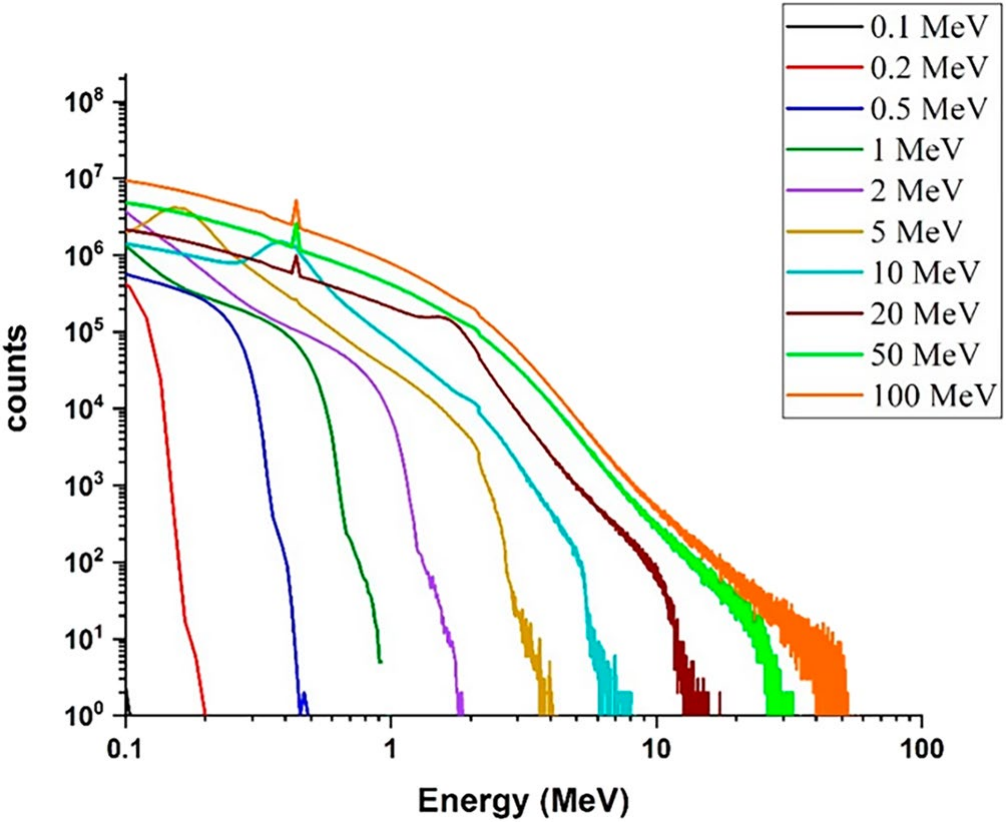
Spectra of deposited energy in the target corresponding to the tangential radiation mode (one degree angle) of single energy electron beam in the presence of a tangential magnetic field (in the x-axis direction) 1 T, to the tungsten target for energies 0.1–100 MeV.

Figure 7 shows the spectrum of energy deposition in the situation where the 1 T magnetic field and incident electron beam are both in the direction of the x axis and are approximately tangent to the target plane (angle 1-degree), while the target plane is perpendicular to the z axis.

**Figure 8:**
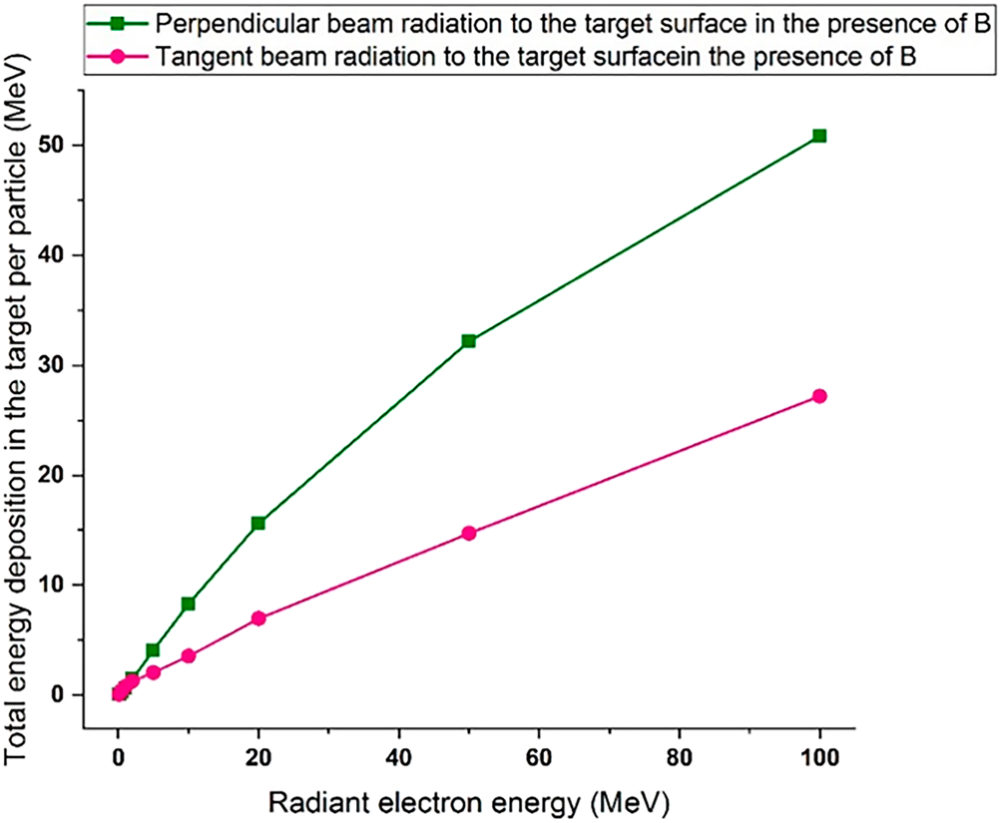
Energy deposited in the target due to the impact of the electron beam perpendicularly and tangentially in the presence of the magnetic field for per electron particle.

By comparing Figs. 3 and 7, it was observed that in the case of tangential radiation of electron beam to the target surface, the amplitude of the energy deposition spectrum of the electron beam in the presence of the field has an increase compared to the case of the absence of the field. Figure 8 shows the values of energy/particle deposition in the case of tangential and perpendicular radiation of the electron beam in the presence of a magnetic field. By comparing Figs. 4 and 8, it can be seen that in the case of perpendicular collision of electrons with the target, the amount of deposited energy per particle in the presence of a magnetic field is not significantly different from the case of the absence of a magnetic field.

Considering that the magnetic field is applied in the direction of the movement of the electron beam, it does not have a significant effect on the movement of primary particles and their interactions inside the target. Also as can be seen in Fig. 8, in tangential radiation to the target (1-degree) by applying a magnetic field, the amount of energy that remains from each particle in the target has increased for different energies compared to the case where there is no field. The reason is that the amount of particles scattered from the surface in the tangent mode is more than the perpendicular radiation of the electron beam. By applying the magnetic field, the scattered particles travel the circular path and have the possibility to return to the target, so the interaction of particles in the tangent state increases with the presence of the field, and after averaging the number of particles, the energy deposition of each particle in the target increases in comparison to the absence of field.

The increase in energy deposition per particle is greater for particles with lower energies, because in this case, the amount of particles reflected from the surface after impact will be greater compared to particles with higher energies, and the magnetic field has a greater effect on the number of reflected particles. By analyzing the obtained data, it is concluded that in the case of electrons hitting the target surface with smaller angles, the magnetic field will have a greater effect on the deposition of energy caused by each particle in the target. In other words, by increasing the angle of incidence and the energy of the electron beam, the effect of the magnetic field on energy deposition will decrease, and this result is in agreement with other references such as reference^38^.

### Contribution of physical processes to the energy deposition spectrum from single-energy electron collisions with the target

This section explores how runaway electrons deposit energy when they collide with a tungsten target. It calculates the energy deposition for different physical processes, studies how these processes reduce the electron's energy upon interacting with the target, and focuses on perpendicular collisions without a magnetic field. The simulation covered reactions like gamma conversion, multiple scattering, bremsstrahlung, ionization, Compton scattering, photoelectric effect, pair production, annihilation, and Rayleigh scattering. This study focused on electron beams with energies of 0.1, 0.2, 0.5, 1, 2, 5, 10, 20, 50, and 100 MeV, oriented perpendicularly to the target surface, without a magnetic field.

**Figure 9:**
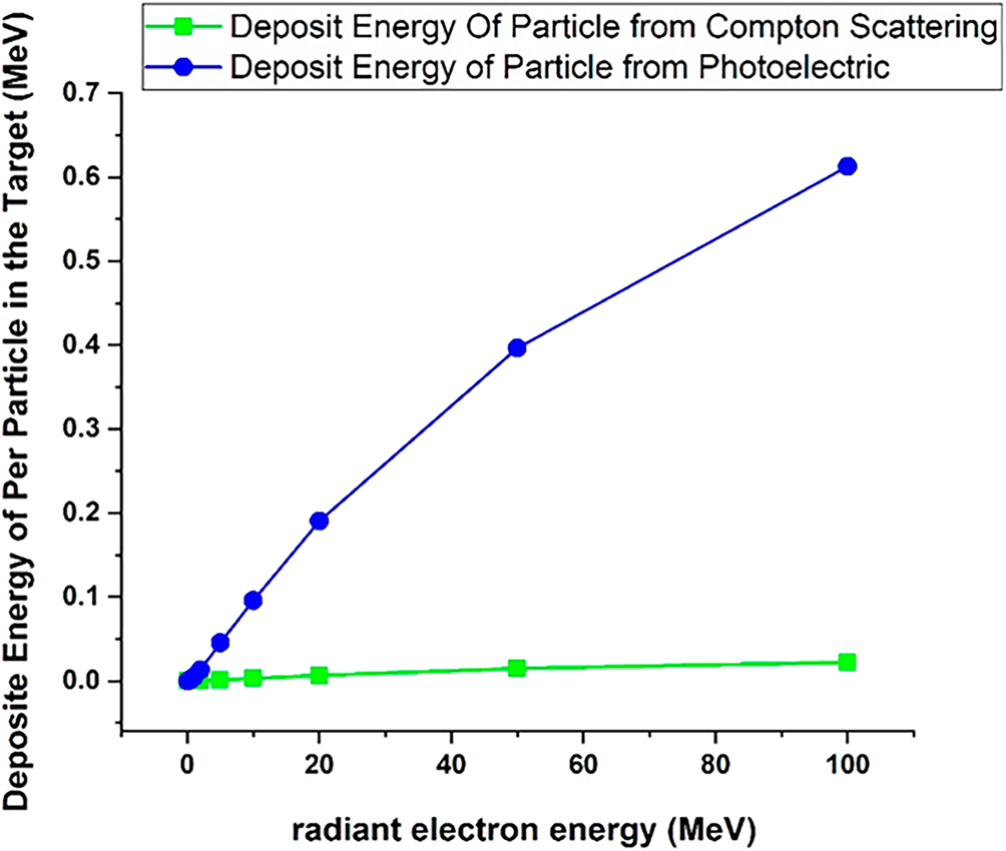
The deposition energy of each incident electron in tungsten target due to photoelectric and compton scattering processes.

Figure 9 presents the energy deposition resulting from the photoelectric and Compton scattering processes for individual incident electrons colliding with the tungsten target. It is evident from the figure that the energy deposited due to the photoelectric effect increases with the energy of the incident electrons. However, it's important to note that the contribution of the photoelectric phenomenon to the total energy deposited by the incident electrons is less than 1% although the amount of deposited energy per particle is significantly lower for Compton scattering.

Simulation results indicated that the energy deposition due to photoelectric reaction occurs only in a few specific energies and the energy spectrum resulting from this process is not a continuous spectrum. Also the energy deposition caused by Compton scattering occurs in only a few specific energies which are in line with the energy of K and L levels of tungsten.

**Figure 10:**
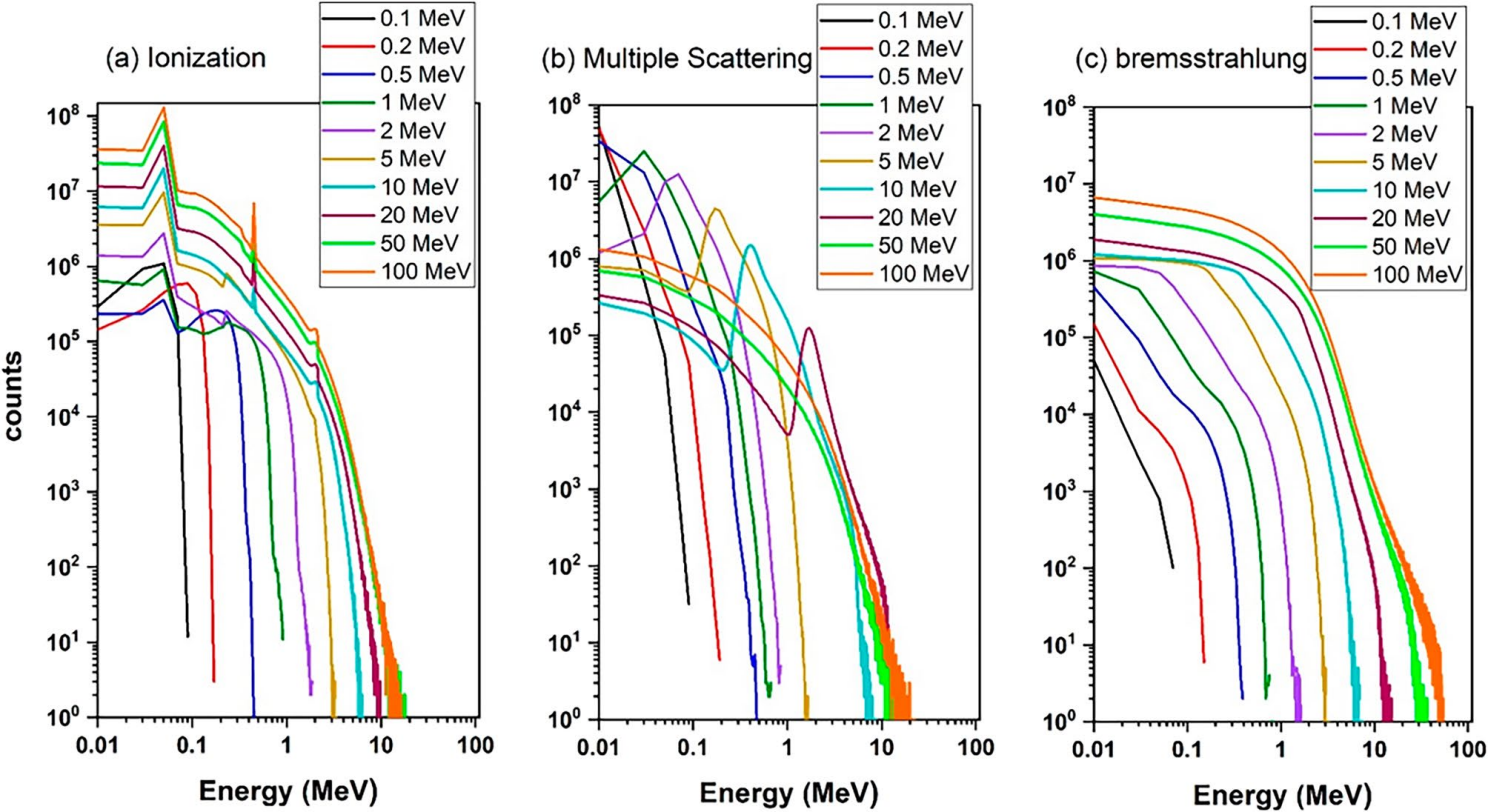
The energy deposited spectrum due to the (**a**) ionization, (**b**) multiple scattering and (**c**) bremsstrahlung phenomena in the tungsten target is related to the perpendicular radiation mode of single-energy electrons without the presence of a magnetic field for energies of 0.1–100 MeV.

Figure 10a displays the energy deposition spectrum resulting from the ionization process during the single-energy electrons collision with the tungsten target. As observed, the energy deposition spectrum related to the ionization phenomenon is a continuous spectrum.

By comparing Figs. 2 and 10a, it is observed that for energies lower than 2 MeV, the energy deposition spectrum resulting is similar to the overall energy deposition spectrum. However, for energies higher than 2 MeV, the width of the energy deposition spectrum from ionization shows a significant difference compared to the width of the energy deposition spectrum from all processes. The simulations demonstrated that ionization plays a major role in electron energy deposition in the target.

Figure 10b presents the electron energy deposition spectrum in the tungsten target due to the multiple scattering process. Notably, in the energy range of 0–2 MeV, an increase in electron energy leads to a widening of the energy deposition range caused by multiple scattering, along with an increase in its probability. However, in the energy range of 5–100 MeV, the increase is less pronounced. This indicates that the contribution of energy deposition due to the multiple scattering process decreases with increasing electron energy.

The reason for this occurrence is that, considering that the thickness of the tungsten target is 10 mm, with the increase in energy, the penetration depth of the particles in the target also increases and a large number of electrons with high energies will be able to pass through the target, so that the amount of collisions with other target particles and the sharing of energy in the target is reduced. Nevertheless, at low energies, due to the fact that the thickness of the target is several times larger than the penetration depth of the particle at those energies, the electron is able to scatter many times and interact with other electrons and particles of the target and transfer its energy. Therefore, for this thickness of 10 mm, the contribution of the energy deposition due to the multiple scattering process in the total energy deposition spectrum for energies higher than 10 MeV shows a significant decrease. The simulation indicated that the thickness of the target is important in the multiple scattering process and if this thickness is increased, the increasing the energy deposition will continue.

Figure 10c shows the changes in the energy deposition of the incident electron due to the bremsstrahlung process in the tungsten target for electrons with different energies.

As can be seen, the range of deposited energy by the bremsstrahlung process is very similar to the range of deposited energy caused by all processes (Fig. 2). This observation highlights the significant contribution of energy deposition attributed to bremsstrahlung in the total energy deposition spectrum, particularly at higher energy ranges. In this diagram, with increasing the energy of electrons, the amount of energy deposition increases. It should be noted that the energy deposition abundance for the bremsstrahlung process is significantly lower, in comparison to ionization and multiple scattering processes, especially at energies less than 0.5 MeV, which indicates the small contribution of bremsstrahlung in the total energy deposition for low energies in Target.

**Figure 11:**
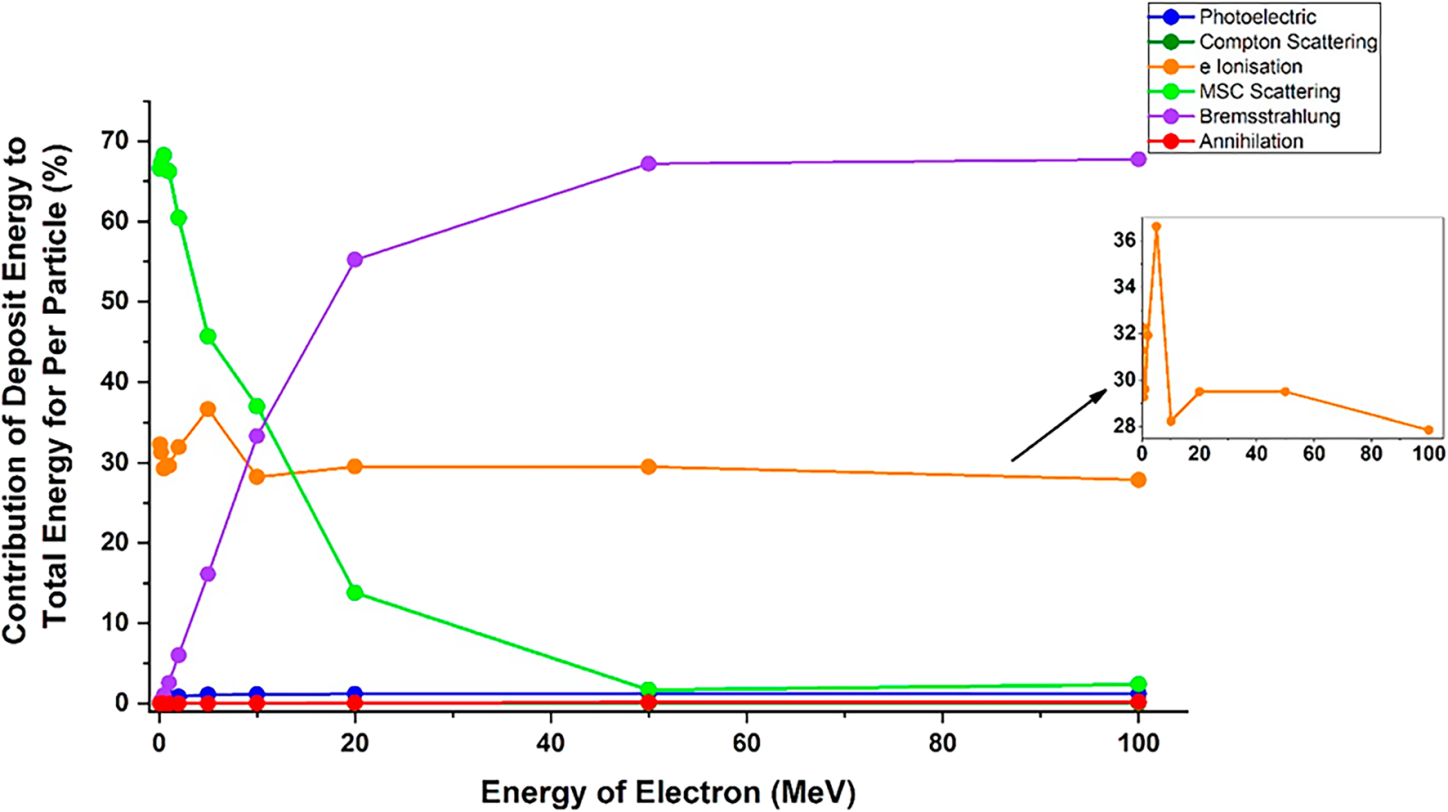
The percentage of deposited energy due to each process per incident electron with specific energies, For better explanation, the percentage of energy deposition contribution graph related to the ionization process is magnified.

In Fig. 11, the contribution of each process in the energy deposition caused by the collision of the runaway electron beam with the tungsten target are shown as curves. As can be seen, in the thickness of 10 mm of the tungsten target, for low energies (less than 5 MeV), the most energy deposition in the target is caused by the multiple scattering and the ionization processes and the bremsstrahlung, Compton scattering and photoelectric have a small contribution to the depositing of the incident electron energy in target. In general, the contribution of Compton and photoelectric processes in the deposition of incident electron energy in the target is not significant compared to the total deposited energy due to all processes, and these two reactions do not have a significant contribution in the deposition of particle energy in the target. At low electron energies, the bremsstrahlung radiation has a very small contribution to the particle energy deposition. But, as the energy of the electrons increases, the contribution of the bremsstrahlung phenomenon to the particle energy deposition in the target increases, so that for electrons with energy of more than 50 MeV, the most energy deposition in the tungsten target is caused by the bremsstrahlung.

It should be noted that for particles with energy from 2 to 100 MeV, pair production and pair annihilation also occur, so that the pair production process does not contribute to the energy deposition, but the effect of the pair annihilation process on energy deposition increases with increasing particle energy. The pair annihilation phenomenon, like the Compton process, has a contribution of less than 1% in the deposition of particle energy in the target.

According to Fig. 11, at low incident electron energies, the ionization process is one of the main processes in the deposition of particle energy in the target, but with increasing electron energy, the contribution of this process to the total energy deposition gradually decreases compared to low energy electrons. The reason for this, as mentioned earlier, is the increase of particles passing through the target at higher energies and therefore the reduction of the ionization process by the primary electrons hitting the target.

**Figure 12:**
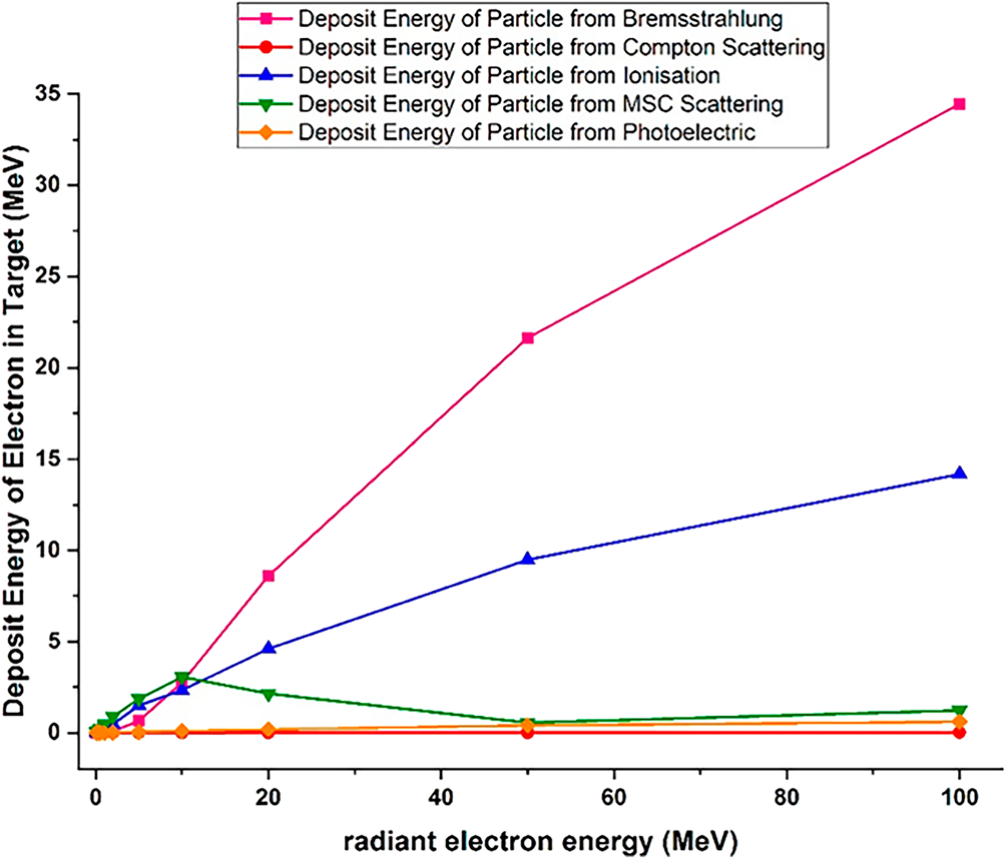
Deposition of residual energy from per incident electron with specific energies in the tungsten target caused by photoelectric, Compton, ionization, multiple scattering and bremsstrahlung process.

In the case of multiple scattering, because high-energy electrons have a smaller collision cross section than lower energy electrons^39^, increasing the electron energy and increasing the penetration depth of electrons inside the target reduces the amount of this interaction and the contribution of this process to particle energy deposition in the target. Figure 12 shows the residual energy deposition of each electron in the tungsten target for all the investigated processes.

**Figure 13:**
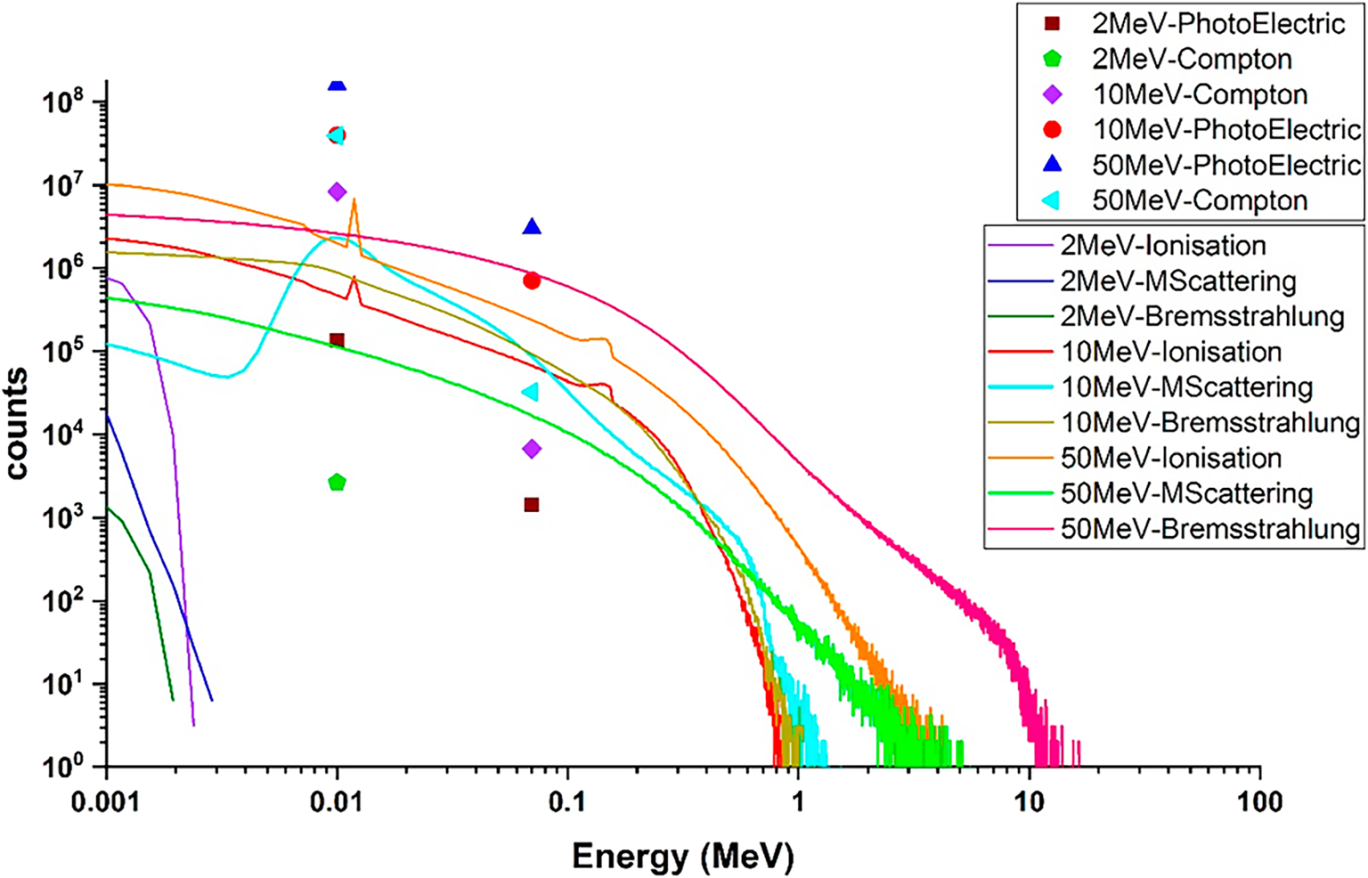
The energy deposited spectrum due to the photoelectric, Compton, ionization, multiple scattering and bremsstrahlung phenomena in the tungsten target is related to the perpendicular radiation mode of five million single-energy electrons to the target without the presence of a magnetic field for energies of 0.2, 10 and 50 MeV.

In order to have a better comparison for the spectrum of energy deposition related to Compton scattering, photoelectric, ionization, multiple scattering and bremsstrahlung processes, for example, three energies of 2 MeV, 10 MeV and 50 MeV were selected for electron beam as low, medium and high energy, respectively. The spectrum of energy deposition caused by the mentioned processes for electrons with these energies is given in a figure which can be seen in Fig. 13.

### Energy deposition resulting from electron collisions within the depth of the target

Previously, in the calculation section, it was stated that by meshing in the target, the amount of energy deposited from the collision of single-energy electron beams caused by all possible physical processes in each mesh was calculated, and thus the amount of particle energy deposition at the depth of the target for different beam energies was obtained.

**Figure 14:**
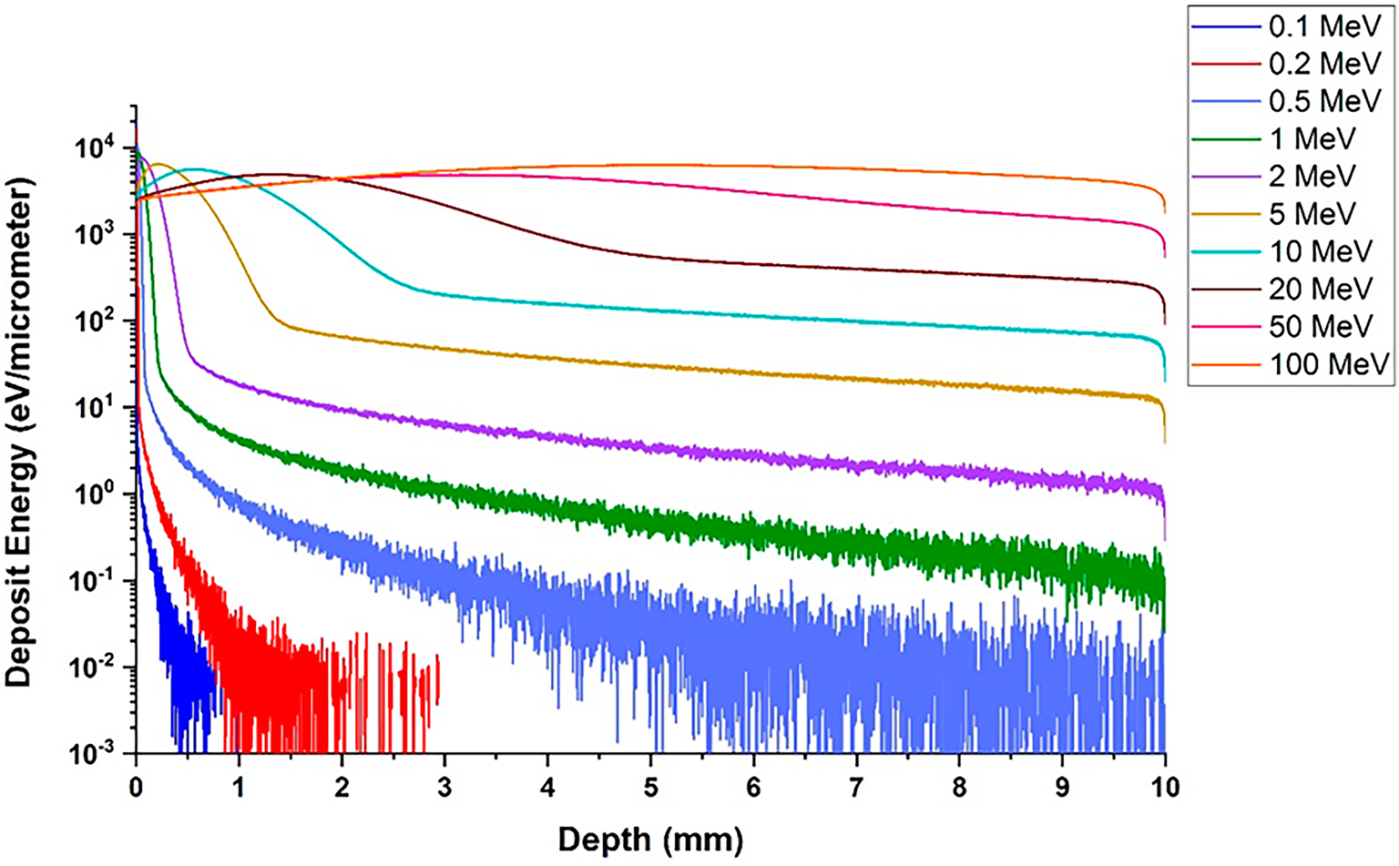
Energy deposition is caused by the interaction of a single energy electron in the depth of the tungsten target per particle.

As can be seen in Fig. 14, with the increase in the energy of the electron beam, the amount of energy deposition in the depth of the target has increased. Upon the impact of the electron beam with energy of 0.1–0.5 MeV, the maximum energy deposition occurs in layers close to the outer surface of the target. In this energy range, as the electron energy increases from 0.1 to 0.5 MeV, this layer moves from the outer surface to the inner layers. By increasing the electron energy more than 10 MeV, as can be seen in the figure, the absorption peak and beam energy deposition in the target is broadened compared to lower energy electrons. In high energies, the incident electrons interact almost uniformly throughout the entire depth of the target. The calculations were performed for electron beams of 5 million particles but finally the figure was drawn per particle. Considering that the thickness of the tungsten wall for ITER and DEMO divertors and DEMO limiters is about 6–13 mm, the level of hazards caused by the deposition of this amount of energy in wall materials and plasma facing components in large tokamaks can be estimated. In this simulation, it was found that runaway electrons with energy of about 100 MeV and above are able to penetrate the entire length of the tokamak wall and plasma facing components and deposit very high energy, and this may lead to significant damage to the wall as well as other equipment behind the wall. In addition, the absorption of this amount of energy in a small and localized area and in a very short period will lead to the deep melting of the wall and its consequences such as failure, cracking and mechanical damage of the wall.

### Investigating the penetration depth of runaway electrons according to the energy of the particles

**Figure 15:**
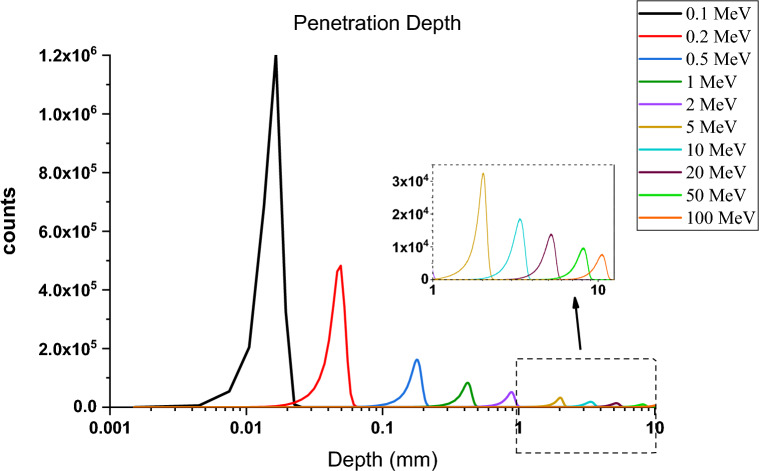
Distribution diagram of electron penetration depth in tungsten target for energies 0.1, 0.2, 0.5, 1, 2, 5, 10, 20 and 50 MeV.

The depth of the electron's penetration in the tungsten target has been investigated in this section. The penetration depth spectrum of a single energy electron beam into the tungsten target in perpendicular radiation mode was computed using simulation by Geant4 code at different energies. Figure 15 shows the penetration depth diagram for incident electrons with energies of 0.1, 0.2, 0.5, 1, 2, 5, 20, 50 and 100 MeV. Considering that at energies less than 1 MeV, the depth of penetration of the electron is up to 0.5 mm, and for higher energies, it increases to 10 mm, for this reason, the calculated values are plotted in logarithm scale. The average value of this spectrum is calculated by software that determines the depth of electron penetration in tungsten and these values are given in Table 1. The average value of electron penetration depth in the tungsten target is completely consistent with the value of electron penetration depth obtained from the NIST Table^40^.

**Table 1:** Electron penetration depth in tungsten for different energies.

Energy (MeV)	0.1	0.2	0.5	1	2	5	10	20	50	100
Penetration depth (cm) In Tungsten from NIST^40^	0.00154	0.00458	0.0168	0.0391	1.01	0.780	0.497	0.321	0.191	0.0835
Penetration depth (cm) In Tungsten	0.00153	0.00455	0.0168	0.0395	1.00	0.773	0.493	0.319	0.190	0.0829

As can be seen in Fig. 15, the distribution of the penetration depth of electrons in the target becomes wider with increasing energy, and their peak height decreases. As a result, its average value also increases. According to Table 1, electrons up to 1 MeV energy can only penetrate up to about 400 microns in tungsten, but with the increase in energy, it can be seen that the depth of electron penetration with 100 MeV energy inside tungsten has increased to about 10 mm. At this distance, it is possible for these electrons to interact and transfer energy, and electrons with energy of 100 MeV and above can pass through the wall with a thickness of 10 mm. From Fig. 15, it can be concluded that according to the diagram of electron penetration depth in tungsten, energy deposition is more localized at low energies because the distribution of penetration depth at these energies is peaked. This issue can increase the energy density deposited in a small area and lead to more serious damage.

### Secondary products produced in the interaction of runaway electrons with the wall of the tokamak and the divertor

**Figure 16:**
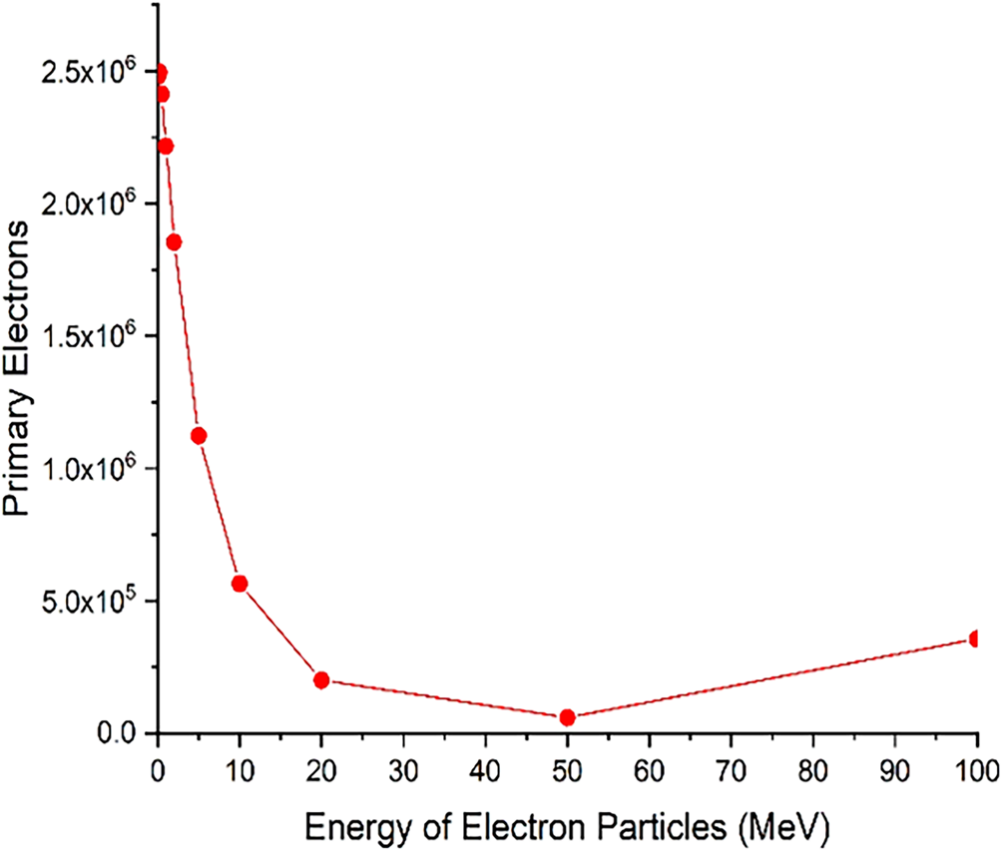
The number of primary electrons that have been ejected from the target for five million electrons hitting the target.

**Figure 17:**
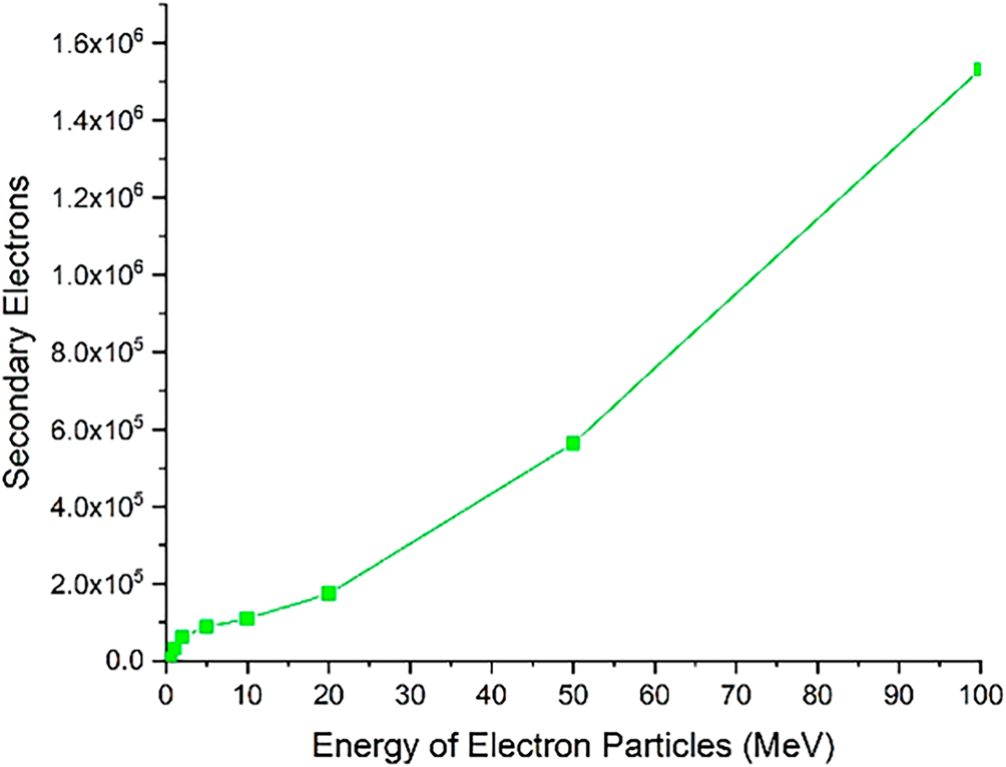
The number of secondary electrons resulting from the interaction of the electron beam consists of five million electrons with the tungsten target that have been ejected from its volume.

**Figure 18:**
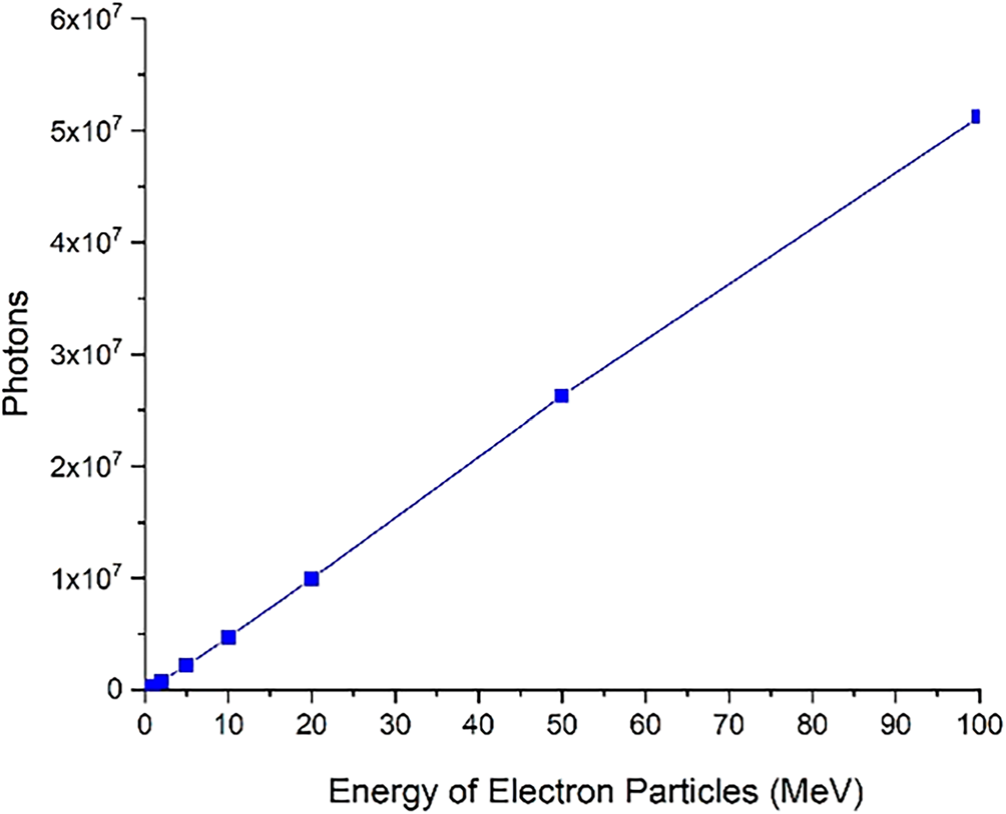
The number of photons produced in the collision of the electron beam including five million electrons with the target that have been ejected from its volume.

**Figure 19:**
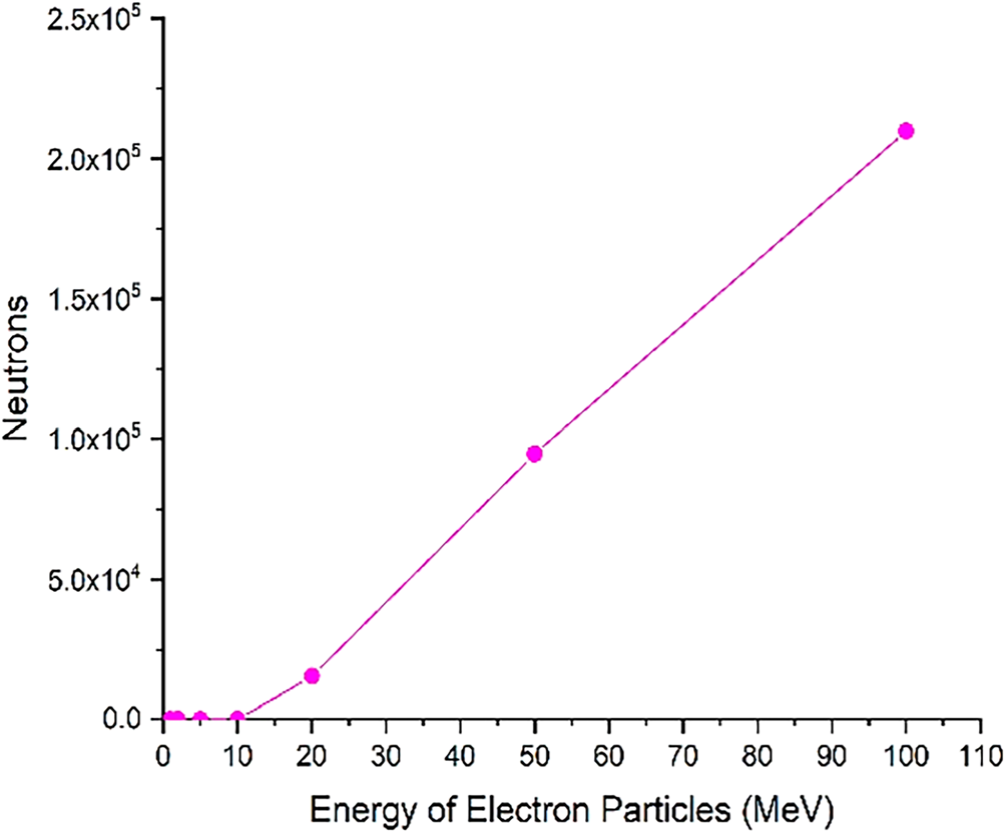
The number of photoneutrons produced during the perpendicular collision of the electron beam containing five million electrons with tungsten that have been ejected from its volume.

As shown in the previous sections, the runaway electrons are able to discharge significant energy when they collide with the tokamak wall and the divertor. However, in the interaction of these electrons with tungsten, a significant amount of secondary products will be produced, which in turn have destructive effects and should be studied and investigated. One of these products is secondary electrons that are produced when energetic runaway electrons collide with the tungsten target and can damage even the tools and equipment behind the wall by passing through the tungsten wall and transferring a large heat load to them. In order to obtain the amount of secondary products, it is necessary to calculate the number of products coming out of the target volume for the impact of electron beams at different energies. First, the perpendicular collision of the electron beam with the target is considered and the departing of the collided electrons and reaction products are presented in Figs. 16, 17, 18, 19. Figure 16 show the number of primary electrons coming out the target volume for different incident electron energies.

As shown in Fig. 16, the number of primary electrons coming out of the total target volume is significant for the electron beam with less than 20 MeV energy. According to Table 1, which shows the penetration depth of electrons inside tungsten, it is expected that at low energies, the penetration rate of particles inside tungsten metal is not high. Therefore, the exiting of primary electrons from the entire target volume indicates the reflection of particles from the target surface. Based on the data presented in Fig. 16, it can be inferred that for electron beams with energies less than 1 MeV striking the tungsten target, the reflection of electrons is highly significant. As the energy of the electron beam increases, the amount of particle reflection from the tungsten surface decreases. Figure 17 shows the amount of secondary electrons ejected from the target volume of tungsten due to the perpendicular collision of the electrons at different energies.

As can be seen in Fig. 17, the number of secondary electrons produced during the electron impact reaction with tungsten leaving the entire target volume increases with growing of electron beam energy. These secondary electrons are the source of particles that make their way outside the tungsten target (which symbolizes the tokamak wall) and can affect other components. Therefore, in the collision of runaway electrons with the tokamak wall, the produced secondary electrons are also an issue that should be considered. In Fig. 18, the number of photons emitted from the total volume of the tungsten target during the perpendicular collision of electrons with the target for different energies of the electron beam is shown. As shown in the figure, the photons produced during the interaction that come out of the target volume will also be high. The number of these photons increases linearly with the energy of the incident electron.

Figure 19 shows the number of photoneutrons produced in the perpendicular collision of a single energy electron beam containing five million particles with a tungsten target for different energies of the electron beam.

The phenomenon of photoneutron emission occurs as a result of the direct reaction of photons with the tungsten nucleus. These neutrons are produced only when runaway electrons with energies of 10 MeV and above collide with the target, and part of these neutrons are able to get out of the target according to the figure, and their energy and the effects of these neutrons should be investigated.

**Figure 20:**
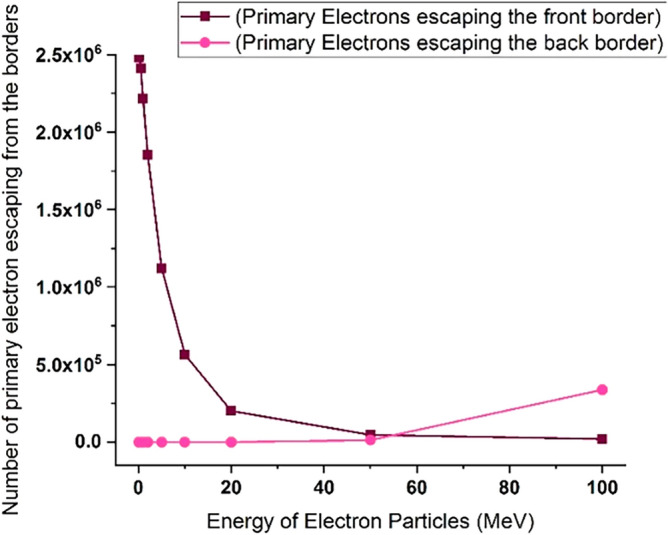
The number of primary electrons emitted from the front and back surface of a 10 mm thick tungsten target for vertical radiation of five million particles to the target.

**Figure 21:**
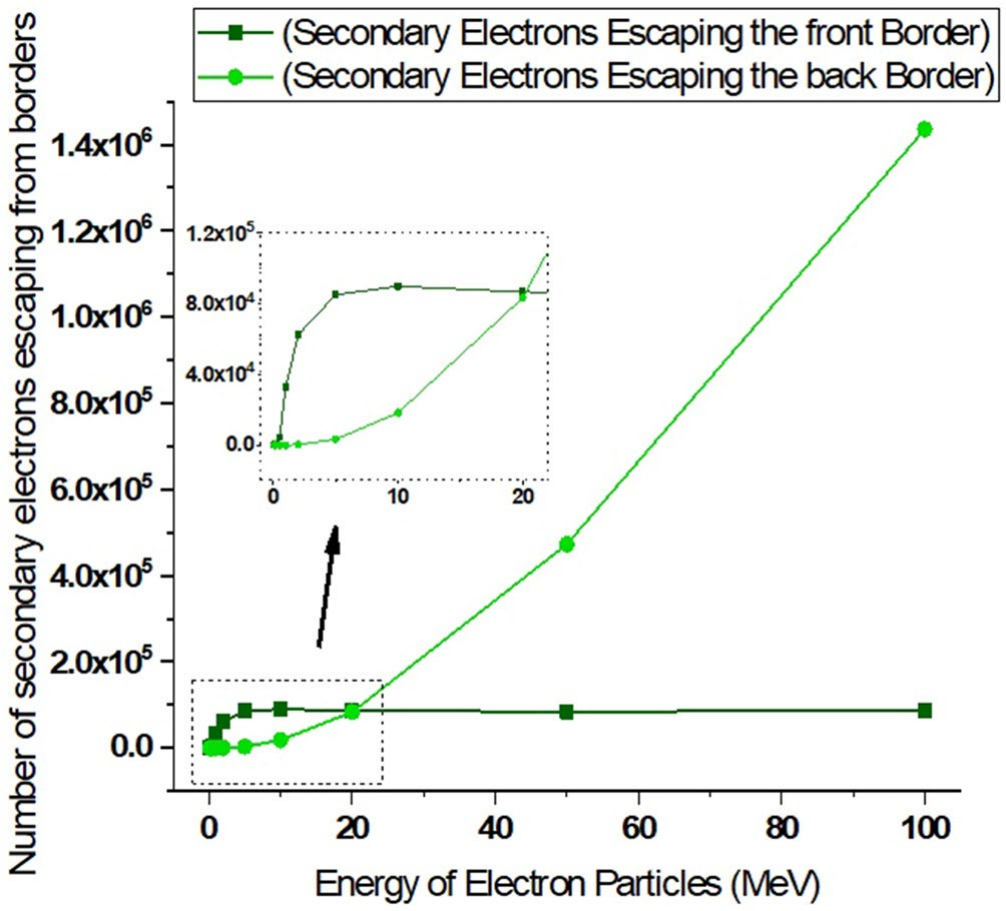
The number of secondary electrons emitted from the front and back surface of a 10 mm thick tungsten target per vertical radiation of five million particles to the target.

**Figure 22:**
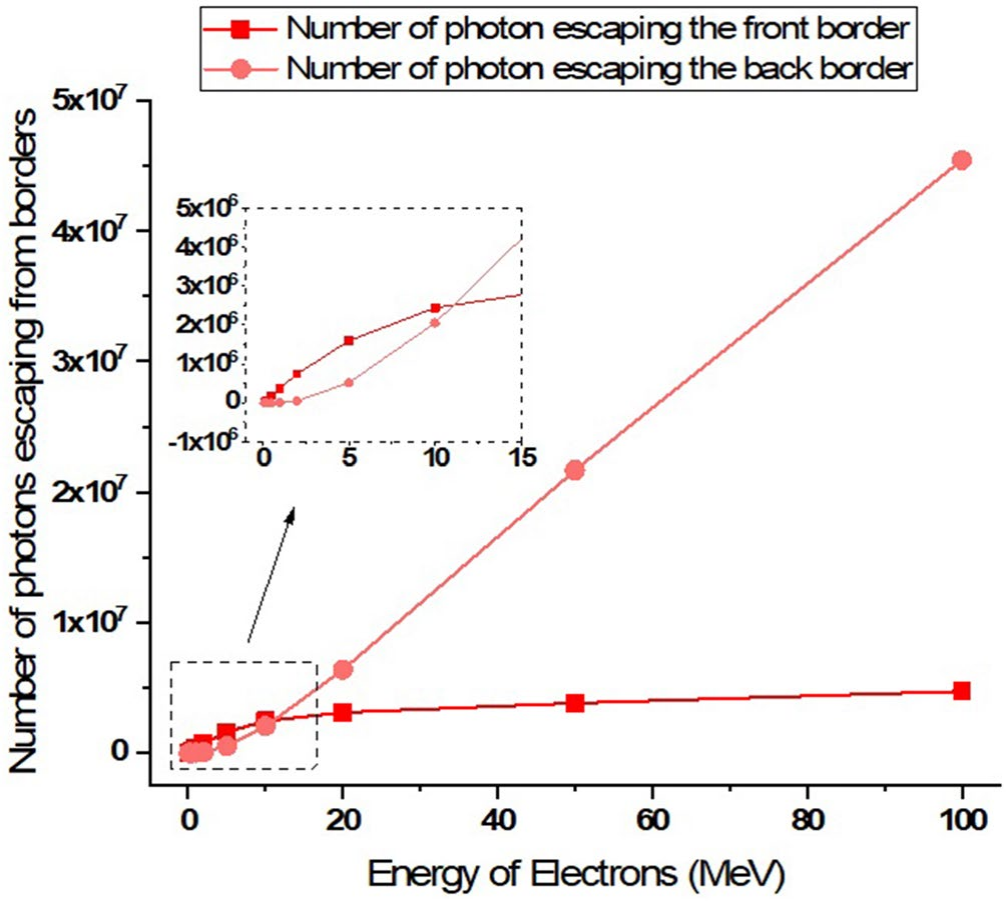
The number of photons emitted from the front and back surface of a 10 mm thick tungsten target per vertical radiation of five million particles to the target.

**Figure 23:**
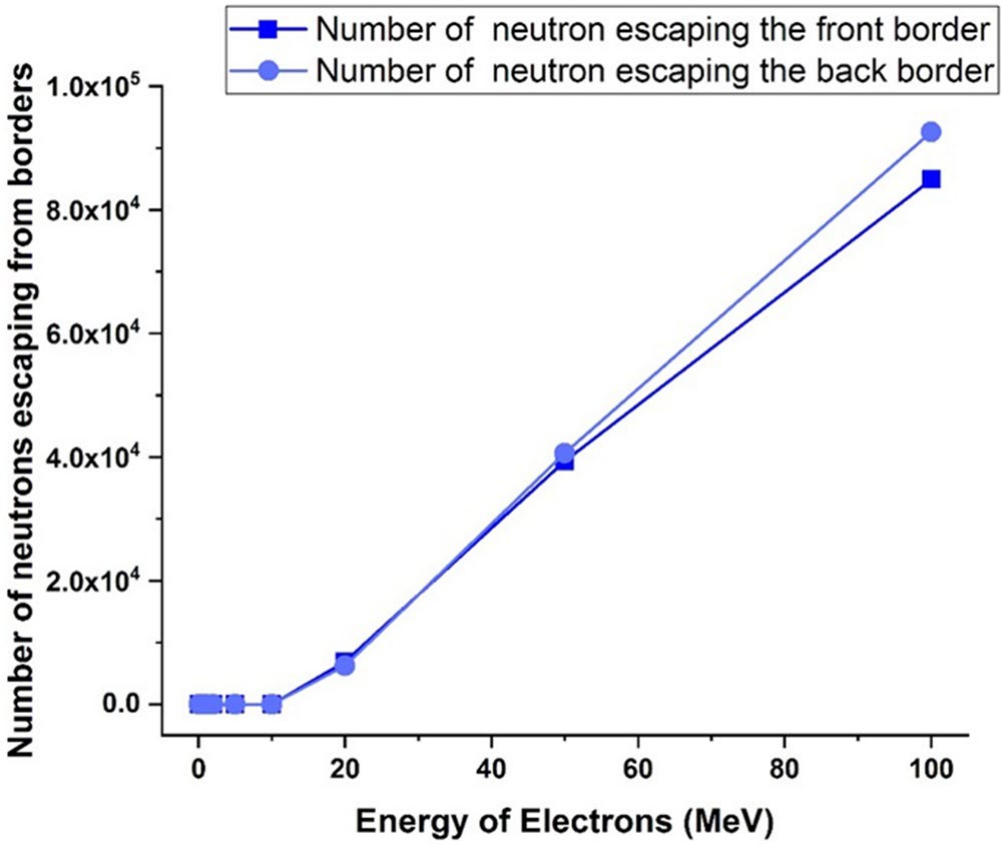
The number of neutrons released from the front and back surface of a 10 mm thick tungsten target for vertical radiation of five million particles to the target.

As inferred from Fig. 16, when electrons with lower energy hit the tungsten target, the number of primary electrons reflecting the target increases. In this part of the calculations, it has been investigated how many of the products produced in the collision of the incident electron beam with the target have left the initial surface and how many have left the final boundary of the target. Using simulation, the number of particles reflected from the initial and final surface of the tungsten target was calculated and presented in the graphs of Figs. 20, 21, 22, 23. Figure 20 shows the number of primary electrons exiting from the initial surface and the final surface of the target.

According to Fig. 20, at energies less than 20 MeV, the number of primary electrons exiting from the front surface of the target is very significant, which indicates the high reflection of these electrons from the surface of the tungsten target. In addition, according to Fig. 20, it is clear that primary electrons with low energies do not actually pass through the end surface of the target due to their small penetration depth in tungsten. But in the case of electrons with higher energy (range 50–100 MeV), the penetration depth of the particles is so high that the primary electrons can be left away from the end of the target, which is clearly visible in Fig. 20. Figure 21 shows the secondary electrons passing through the front surface and the back surface of the target. According to Fig. 21, in the case of incident electrons with energy less than 10 MeV, the secondary electrons are being mostly exited from the front surface of the target.

As can be seen in Fig. 21, for 5 million primary incident electrons with energy 100 MeV that hit the 10 mm thick tungsten target with 100 MeV energy perpendicularly, about 1.5 million secondary electrons leave the end boundary of the target. These electrons are energetic and able to damage the tools and equipment behind the wall and even cause stress and corrosion by interacting with the materials of this equipment. Therefore, the collision of runaway electrons with the tungsten wall produces a source of secondary electrons that are able to pass through this wall.

Figure 22 shows the number of photons ejected from the initial surface and back surface of tungsten target, as the single-energy electrons collide with the target at different energies of the electron beam. Photons are one of the most important reaction products of the collision of runaway electrons with the tungsten target, and these photons have the ability to interact with other materials that contain the wall. According to Fig. 22, as the incident electron energy increases, the amount of photons emitted from the front surface of the wall and its back surface increases. In the collision of electrons with energy less than 1 MeV, the photons exiting from the front and back of the target are not significant compared to higher energies. Therefore, the runaway electrons hitting the tungsten wall act as a source of photons that exit both the front surface and the back surface of the target and are able to affect and interact with other equipment outside the wall.

Figure 23 shows the number of neutrons emitted from the initial and end side of tungsten target for perpendicular collision of electrons at different energies. According to the figure, neutrons are also products of the interaction of high-energy electrons with the tungsten wall, which are produced for electrons with energy of more than 10 MeV and can emit from the tungsten wall. The release of neutrons from both the front surface and the end boundary of the wall are significant. The energy of outgoing neutrons can be used to estimate the effect of possible interactions with instruments and equipment outside the wall.

**Figure 24:**
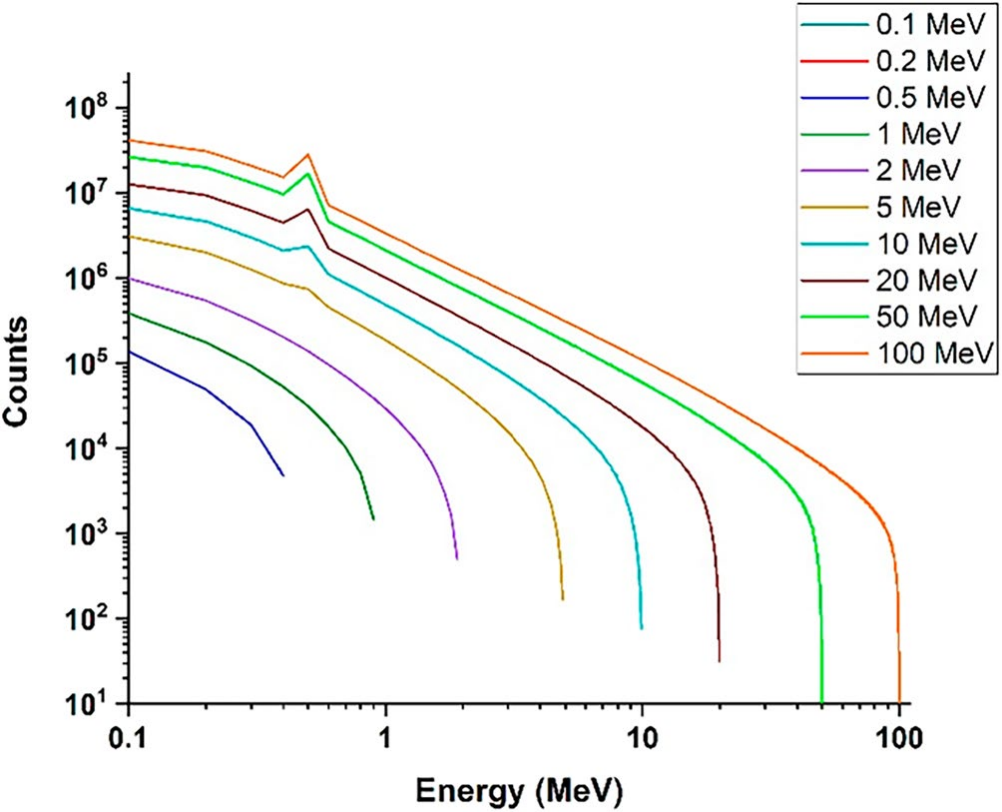
The energy spectrum of photons produced in the perpendicular collision of a single energy electron beam containing five million particles with surface of tungsten target for energies 0.1–100 MeV.

**Figure 25:**
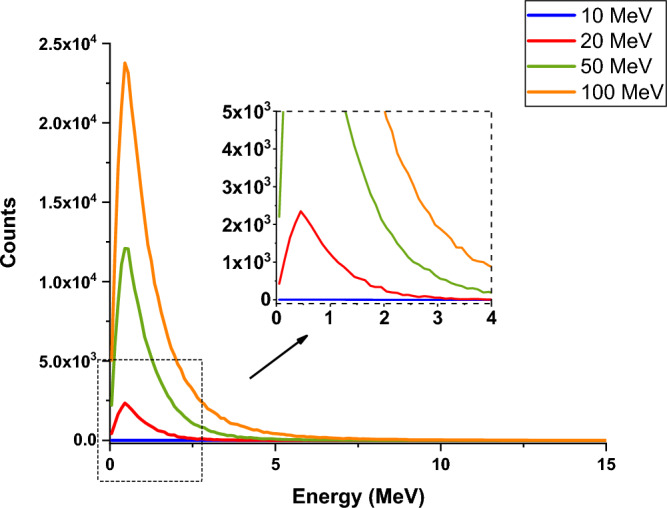
Energy spectrum of neutrons produced in the collision reaction of the electron beam containing five million particles with the tungsten target.

In order to investigate the effects of secondary products produced on equipment and devices outside the target, it is necessary to determine their energy and estimate their potential effects and damages. Using simulation, the profile of the energy spectrum of photons and neutrons produced in the reaction for collision electrons with different energies is calculated. The resulting energy spectra are presented in Figs. 24 and 25.

According to Fig. 24, the energy of the photons produced in the collision of an electron beam with energies of 0.1–100 MeV increases with the increase of the energy of the beam, and for the electron beam with an energy of 100 MeV, photons with an energy near to 100 MeV will also be produced. Although the abundance of these photons is low, they are capable of producing photoneutrons. The threshold energy for the production of photoneutrons in tungsten is 8 MeV, and due to the energy of the photons produced in the collision of the electron beam, the probability of producing photoneutrons will be very high. In addition, the produced high-energy neutrons also leave the target and are able to react with other tokamak equipment and tools.

As can be seen from Fig. 25, with the increase of electron energy, the energy and frequency of neutrons produced due to the impact of electrons on the tungsten target increases. Production of neutrons occurs during the collision of electrons whose energy is in the range of 10–100 MeV, and for electrons with lower energies, neutron production does not occur. The average energy of neutrons produced for a 100 MeV electron beam is about 1.3 MeV, which indicates the production of fast neutrons. As shown in Fig. 23, for electron energies higher than 20 MeV, a large number of produced neutrons will exit the 10 mm thick tungsten target wall. If the production of neutrons occurs at early target intervals, there is a possibility of slowing down and thermalization for these neutrons, which in this case are able to create the process of neutron activation in the wall.

**Figure 26:**
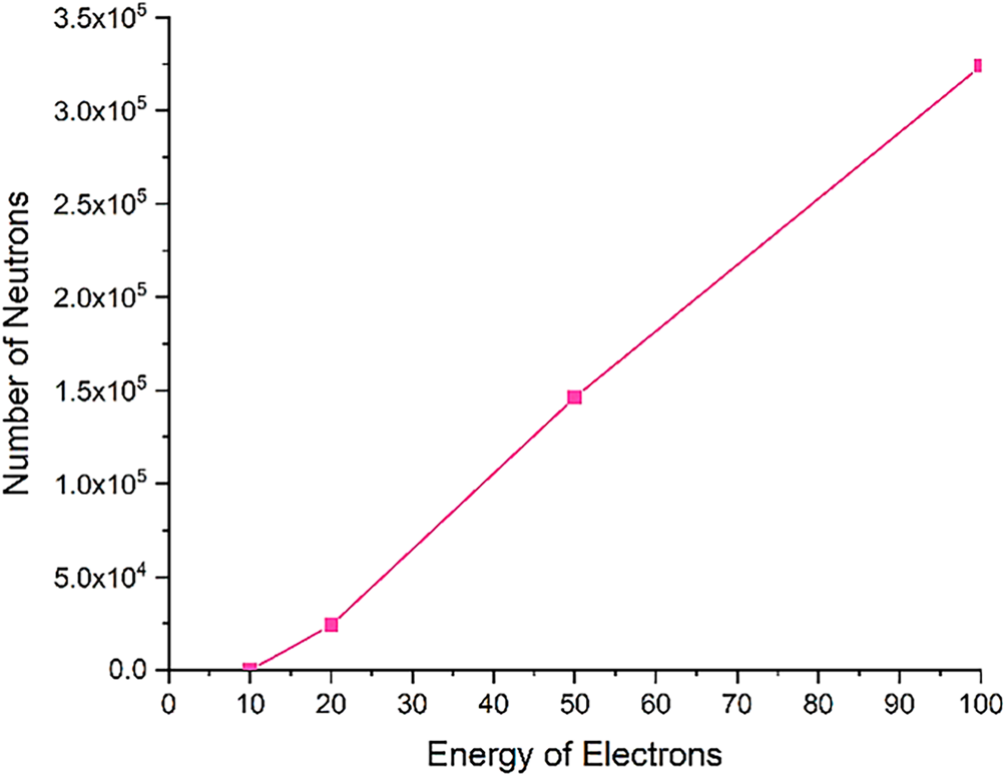
The number of neutrons produced in the interaction of a runaway electron beam containing five million particles with a tungsten target.

The results of simulation with Geant4 showed that during the collision of electrons with energies of 20 MeV and above, the process of neutron capture that causes activation would also occur, which will increase with increasing the energy of the electron beam and its destructive effects will increase. Table 2 shows the number of neutrons produced in the interaction of runaway electrons with the tungsten wall and the number of neutron capture per 5 million particles hitting the target. In addition, the number of neutrons produced during the interaction of runaway electrons and tungsten target is shown in Fig. 26.

**Table 2:** The number of produced neutrons and the resulting reactions.

Electron energy (MeV)	Number of neutron capture reaction	Number of produced neutrons
10	0	38
20	70	24,161
50	445	146,365
100	1019	323,971

### Transport profile of primary and secondary runaway electrons in the tungsten wall of the tokamak

**Figure 27:**
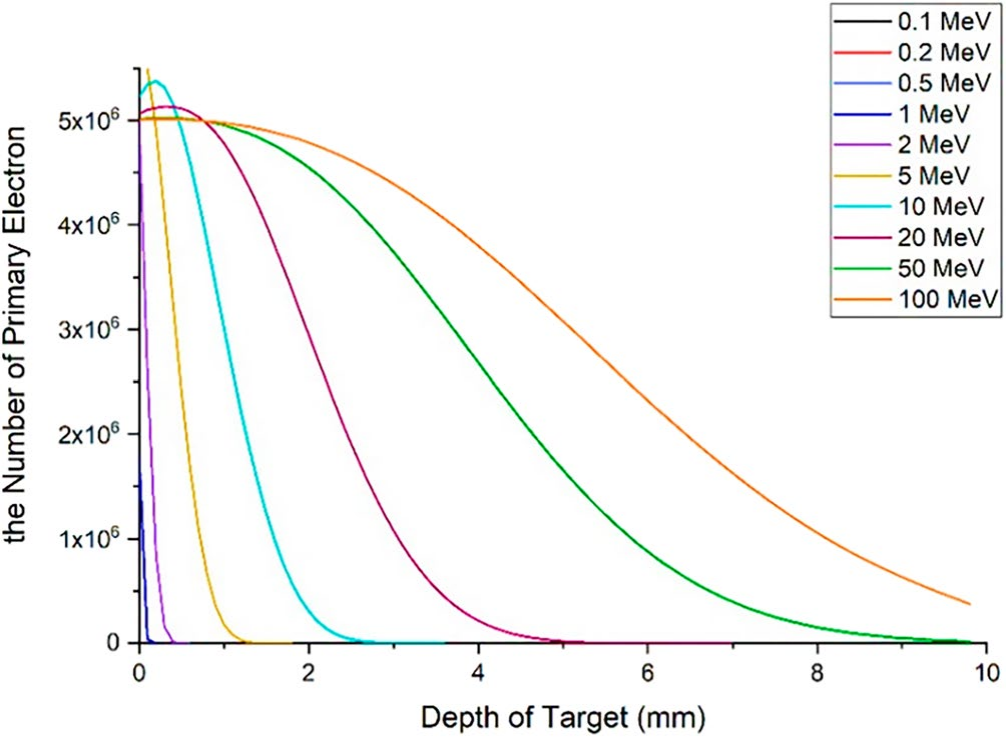
Primary electron transit profile (including five million incident electrons) in the depth of the tungsten target.

**Figure 28:**
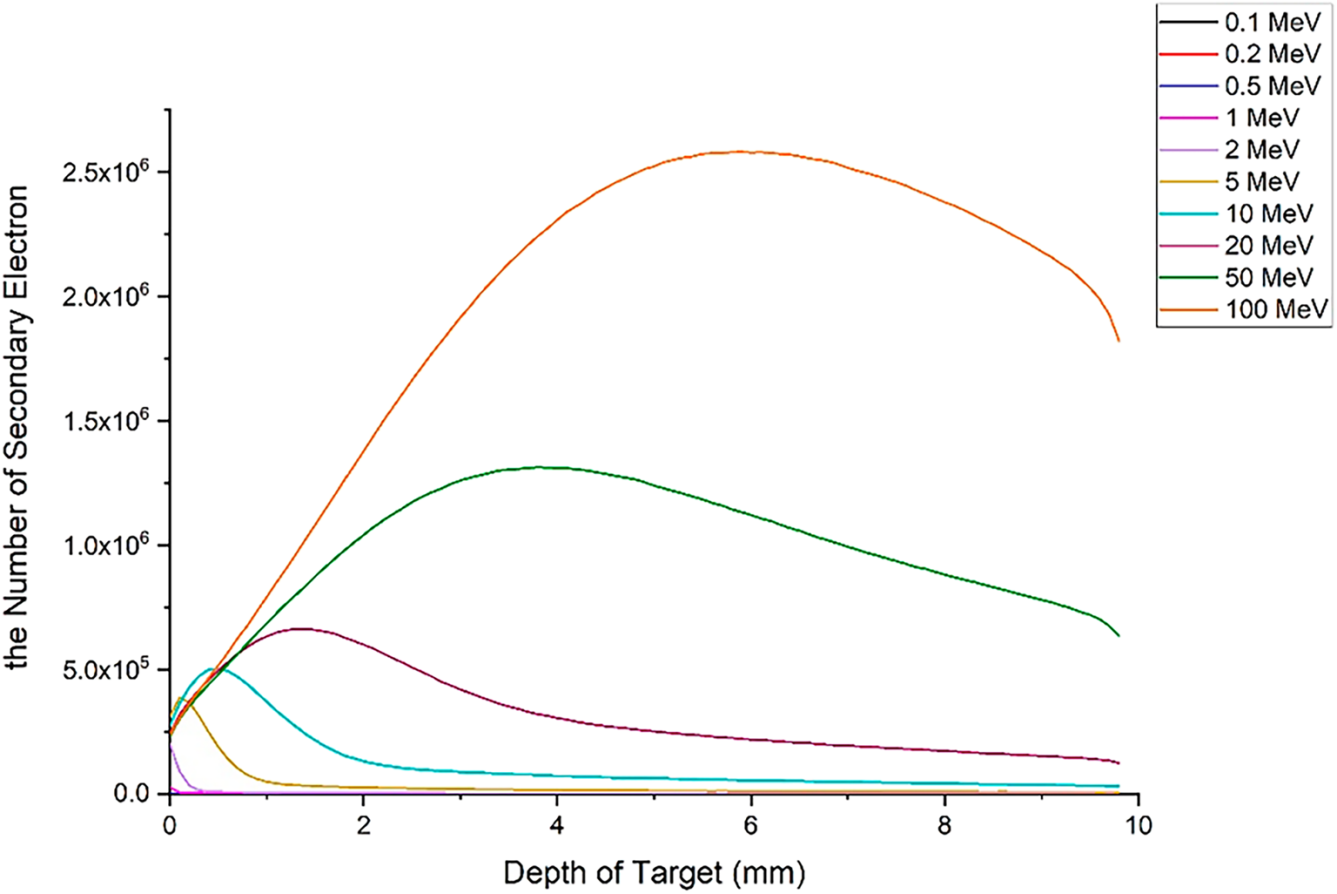
Secondary electron transit profile (caused by the impact of five million electrons on the target), in the depth of the tungsten target.

As described earlier, most primary and secondary electrons are able to penetrate the target and reach the other side. In this section, the passage profile of the primary and secondary electrons produced during the interaction at the depth of the target is calculated using the simulation with the Geant4 code. For this purpose, the tungsten target, which has a thickness of 10 mm, was divided into plates with a thickness of 1μm and was vertically irradiated with 5 million electrons at different energies. Then, the presence of electrons during the interaction was calculated in each of these layers, and finally, the profile of the passage of primary and secondary electrons for this state was obtained from the collision of the electron beam with the target, according to Figs. 27 and 28.

According to Fig. 27, electrons with energies of 0.1, 0.2, and 0.5 MeV do not pass through the layer with a thickness of 0.1 mm and interact in the same layer, and according to the penetration depth of electrons according to Table 1, this result is expected. As can be seen in Fig. 27, for an electron beam with energy 100 MeV, the primary electrons population do not reach zero even after passing through the back surface of the target and passing through it.

Figure 28 shows the transport profile of the secondary electrons produced in the collision of the primary electron beam with tungsten. The number of secondary electrons resulting from the impact of the 0.1, 0.2 and 0.5 MeV electron beam on the target is very small and therefore cannot be clearly seen in the diagram of Fig. 28. With the increase in the energy of primary electrons, the secondary electrons produced during the interaction with tungsten have increased and many of these electrons are emitted from the target. These electrons themselves are considered as a source of electrons that are able to collide with the equipment behind the wall and transfer their energy to them and create mechanical and thermal stresses in this equipment. Additionally, as was already noted, when designing the tokamak wall and divertor, it is important to take into account the generation of runaway electron secondary products and the identification of their consequences.

## Conclusion

In this study, using a simulation with the Geant4 code, a tungsten target with a thickness of 10 mm was exposed to single-energy electron radiation with an energy range of 0.1–100 MeV and various investigations were carried out on the penetration depth of the incident electrons and the spectrum of their energy deposition in the target. Moreover, the effect of various physical processes on the amount of particle energy deposition was studied.

Based on the obtained results, it was found that the penetration depth of the electrons in the tungsten target increases with the increase of the energy of the incident electrons. In addition, with the increase in the energy of the electrons, the range of the particle energy deposition spectrum and the abundance of the deposited energies will also increase. In the investigation conducted for the radiation of runaway electrons with different angles to the target, it was found that with the increase of the impact angle of the incident electrons, the amount of energy deposited in the target and the range of energy deposition spectrum for each particle increases. The calculation results showed that the application of the magnetic field will increase the energy deposition of each electron in the target, and this increase will be much greater at lower angles and lower electron energy values. By applying a magnetic field, the highest change and increase in the energy deposition caused by each incident electron was observed at energies less than 1 MeV and in the tangential radiation mode, and in the case of perpendicular collision, by applying a magnetic field, the amount of energy deposited in the tungsten target per incident electron did not change significantly, which is in agreement with the results of reference^28^.

During the collision of electrons with the target, the reactions that mainly cause the deposition of electron energy in the target are ionization, multiple scattering and bremsstrahlung. Pair production, Compton scattering and photoelectric processes also cause energy deposition in the target to a very small extent. In the collision of electrons with energies less than 5 MeV, multiple scattering reactions and ionization are the two main effective processes in energy deposition in the target, and bremsstrahlung radiation has a very small contribution to the energy deposition. However, with the increase in the energy of the incident electron, the contribution of multiple scattering and ionization processes to the energy deposition is reduced, and the bremsstrahlung process becomes the most effective reaction in the deposition of energy. The process of pair annihilation will occur for electrons with energy of 2 MeV and above, and the contribution of this process to energy deposition, like the photoelectric and Compton phenomena, is small, but it will increase with the increase of the electron energy.

The particle energy deposition profile in the depth of the tungsten target with perpendicular electron beam radiation showed that the location of the maximum energy deposition in the target reaches from 1μm for a 0.1MeV electron to 0.6mm for a 10MeV electron. This means that with the increase in the energy of the incident particle, the place of maximum energy discharge in the target is transferred to the inner layers. Also, with the increase of incident electron energy, the deposited energy peak has become wider and its height has decreased. For electrons higher than 10 MeV, the energy deposition profile remains relatively constant throughout the entire target depth. In the case of a 100 MeV electron, this corresponds to an approximate deposition of 10 keV/μm along the entire length of the wall, representing the energy imparted by a single electron to the tungsten wall. Given that this energy deposition occurs rapidly and is localized on the wall, a substantial number of runaway electrons can potentially melt the entire 10mm thickness of the tungsten wall. This result indicates the severity of the destructive effects of runaway electrons on the tungsten wall of the tokamak.

Furthermore, the conducted studies and simulations in this research encompass other significant results as follows:

1. In addition to the energy deposition of the primary electrons striking the target, several products are produced as a result of the interaction of electrons with the target, a significant part of which can leave the initial or final surface of the target. One of these products is photons, whose number and energy increase with the increase of incident electron energy. For example, in the collision of 100 MeV electrons with tungsten, the energy of the produced photons will reach near 100 MeV, and these high-energy photons can create unplanned consequences behind the tokamak wall.
2. Secondary electrons result from the interaction between incident electrons and the target. The number of these electrons rises with the energy of the incident particles. Essentially, when primary electrons collide with the tungsten wall, they become a source of secondary electrons capable of escaping the wall. This phenomenon has the potential to impact and potentially damage other equipment and devices positioned behind the wall.
3. Neutrons are one of the most important products of the interaction of runaway electrons with the tungsten target. In the interaction of produced photons with threshold energies of 10 MeV and higher, with the tungsten target, photoneutrons are produced which are in the energy range of fast neutrons.
4. One of the important results of this study is that upon the collision of runaway electrons with a 10mm thick tungsten target, certain primary electrons with energy of 10MeV and above can pass through the target without depositing significant energy, potentially causing damage to the equipment behind the target.

## Data Availability

The authors confirm that all codes written in Geant4 environment and all data obtained from simulations in this manuscript will be made available to the scientific community upon request to the corresponding author of the paper.
